# Cell-Wall-Degrading Enzymes-Related Genes Originating from *Rhizoctonia solani* Increase Sugar Beet Root Damage in the Presence of *Leuconostoc mesenteroides*

**DOI:** 10.3390/ijms23031366

**Published:** 2022-01-25

**Authors:** Rajtilak Majumdar, Carl A. Strausbaugh, Paul J. Galewski, Rakesh Minocha, Christopher W. Rogers

**Affiliations:** 1 Northwest Irrigation and Soils Research, United States Department of Agriculture, Kimberly, ID 83341, USA; paul.galewski@usda.gov (P.J.G.); christopher.w.rogers@usda.gov (C.W.R.); 2 Northern Research Station, USDA Forest Service, Durham, NH 03824, USA; rakesh.minocha@usda.gov

**Keywords:** sugar beet, pathogenesis, pectin lyase, polygalacturonase, cellulase, mRNAseq, C/N ratio

## Abstract

Sugar beet crown and root rot caused by *Rhizoctonia solani* is a major yield constraint. Root rot is highly increased when *R. solani* and *Leuconostoc* *mesenteroides* co-infect roots. We hypothesized that the absence of plant cell-wall-degrading enzymes in *L*. *mesenteroides* and their supply by *R. solani* during close contact, causes increased damage. *In planta* root inoculation with or without cell-wall-degrading enzymes showed greater rot when *L. mesenteroides* was combined with cellulase (22 mm rot), polygalacturonase (47 mm), and pectin lyase (57 mm) versus these enzymes (0–26 mm), *R. solani* (20 mm), and *L*. *mesenteroides* (13 mm) individually. Carbohydrate analysis revealed increased simpler carbohydrates (namely glucose + galactose, and fructose) in the infected roots versus mock control, possibly due to the degradation of complex cell wall carbohydrates. Expression of *R. solani* cellulase, polygalacturonase, and pectin lyase genes during root infection corroborated well with the enzyme data. Global mRNAseq analysis identified candidate genes and highly co-expressed gene modules in all three organisms that might be critical in host plant defense and pathogenesis. Targeting *R. solani* cell-wall-degrading enzymes in the future could be an effective strategy to mitigate root damage during its interaction with *L*. *mesenteroides*.

## 1. Introduction

Sugar beets (*Beta vulgaris* L.) contribute to 55% of the total sugar produced in the United States (https://www.sugar.org/about/us-industry/, accessed on 5 September 2021). Sugar beet biotic stressors, such as fungi and viruses, are some of the major yield constraints that limit sugar production [1,2,3,4]. Resistance to these pathogens is highly limited due to the lack of appropriate genetic resources [1,5]. When available, resistance is usually controlled by a few genes that are being potentially compromised by the evolving pathogens, or if resistance is controlled by many genes (quantitative trait) it becomes highly challenging and time consuming to introgress resistance into the commercial hybrids. In many cases, genetic resistance comes with a trade-off of reduced sugar yield. The future use of cutting-edge molecular biology tools, such as RNA interference (RNAi) and CRISPR-Cas, could help improve host resistance, but this requires the identification of potential target genes in pathogens that are highly critical in controlling pathogenesis and the development of disease symptoms.

Rhizoctonia crown and root rot (RCRR) in sugar beet is caused by the fungus *Rhizoctonia solani* Kühn, and it is a major problem in the United States and around the world. The fungus can attack the sugar beet at early seedling stages by targeting hypocotyls below the soil causing damping-off, and can attack mature plants at later root development stages. The symptoms are underground roots exhibiting black root lesions, including large mycelium-filled cracks [2]. This disease can contribute to a >50% yield-loss of sugar beet in the field, reduce sucrose content in the roots, and negatively affect factory processing [2,6]. *Rhizoctonia solani* strains in the soil form a species complex and are further classified into subgroups, anastomosis groups (AGs), and intraspecific groups (ISGs). The predominant *R. solani* strain in Idaho is AG-2-2 IIIB, which causes the most rot in mature sugar beet roots [2]. In southwestern Idaho, RCRR can cause a total crop loss when combined with other abiotic and biotic stressors, such as difficult furrow irrigation conditions, warmer and longer growing seasons, and curly top and rhizomania infections [2,7,8]. In the United States, sources of genetic resistance to RCRR in sugar beets are highly limited [9] and are primarily dependent upon the resistance source derived from the sugar beet germplasm FC709-2 [10]. Therefore, additional sources of rhizoctonia resistance are highly desirable.

Another important factor that can increase sugar beet root rot symptoms is the presence of *Leuconostoc* sp., a gram-positive bacterium in the soil [8,11]. Besides soil, *Leuconostoc* can occupy other diverse materials, including fermented vegetables, plant surfaces, wine, and manure to name a few [2]. Furthermore, the association of *Leuconostoc* spp. with fermented sugar beet roots may not be unexpected, as the bacterium is known to be involved at the early stages of the fermentation processes [11]. Typically, the dry black rot of sugar beet roots is associated with *R. solani* infection, primarily on root surfaces and the adjacent underlying tissues, whereas wet and fermented-smelling root rot are associated with bacteria and yeast and extends deeper inside the root tissues. The increase in bacterial growth in rotting sugar beet roots can restrict *R. solani* growth, possibly due to the highly acidic pH of the infected and adjacent root tissues [12]. The predominant strain of *Leuconostoc* isolated from rotting sugar beet roots has been primarily *L. mesenteroides* subsp. *dextranicum* (Beijerinck) Garvie [8,11].

Earlier work from our lab has demonstrated that *R. solani*-mediated root rot in sugar beets is highly increased when *Leuconostoc mesenteroides* (gram + bacteria) is present in close association with *R. solani* in the soil [8]. The fungus, though limited in damaging the outer 3–5% of the sugar beet root mass, can lead to a synergistic interaction that can cause >70% of the root mass to be rotted when in the presence of *L. mesenteroides* [3,11,13]. In fact, *L. mesenteroides* alone in the soil has minimal impact on sugar beet roots. The mechanism by which *R. solani* contributes to increased root rot symptoms is unknown. As *L. mesenteroides* lacks plant cell-wall-degrading enzymes (PCWDEs), we therefore hypothesized that *Leuconostoc* utilizes *R. solani*-derived PCWDEs to overcome the barrier of the plant cell wall and enter inside the root cells. This is followed by a rapid multiplication of the bacteria, thereby increasing root rot symptoms. Through a combination of tools involving the use of purified enzymes, an analysis of cell-wall-degraded carbohydrates and their impact on total C and N in the cells, and a global mRNAseq analysis at early root infection stages (24 h, 48 h, 72 h), we investigated the complex 3-way interaction between sugar beet–*R. solani*–*L. mesenteroides*. Our work demonstrates the specific roles of *R. solani*-derived PCWDEs in increasing root rot under natural conditions. Using a systems biology approach, we identified differentially expressed genes and gene clusters that were highly co-expressed and which might be critical in early *R. solani* and *L. mesenteroides* pathogenesis, as well as in sugar beet host defense response against these pathogens. This is the first report demonstrating the mechanism by which *R. solani* interacts with *L. mesenteroides* during sugar beet root infection and increase root damage.

## 2. Results

### 2.1. Cell-Wall-Degrading Enzymes Increased Root Rot Symptoms When Combined with Pathogen Inoculations

**Figure 1:**
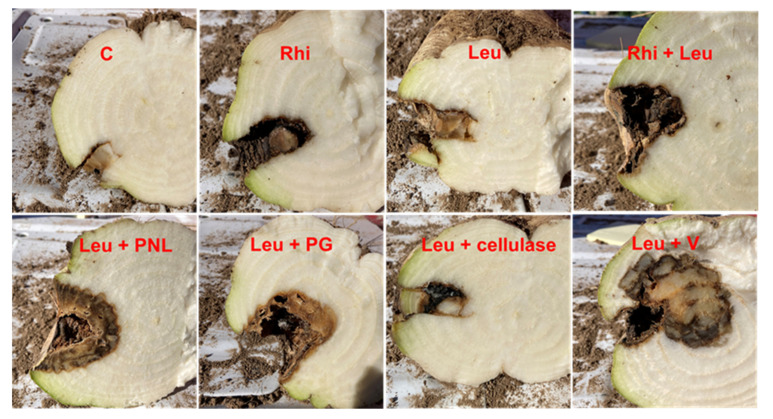
Root rot symptoms are increased in the *Rhizoctonia solani* (Rhi) + *Leuconostoc mesenteroides* (Leu)-infected samples or Leu + PNL/PG/V-treated samples vs. mock control (C). Cross sections of sugar beet roots at 6 weeks post-inoculation in the field.

The data from the two field studies were pooled for analyses (Table 1) since these were not significantly different (*p* = 0.1887), interactions were not significant (*p* = 0.1471 to 0.8664), and their variances were homogeneous (*p* = 0.6036). When compared across all treatments (Table 1, Figure 1), treatments with *L. mesenteroides* had more (*p* < 0.0001) rot (40 mm) than treatments without *L. mesenteroides* (20 mm). When *L. mesenteroides* and *R. solani* were inoculated individually, limited rot occurred (13 and 20 mm, respectively), but significantly more rot (33 mm) occurred when combined. In Table 1, when the enzymes were inoculated individually and in combination with *L. mesenteroides*, rot was higher in all comparisons versus the enzymes without *L. mesenteroides* (rot with and without *L. mesenteroides*): pectin lyase (PNL; 57 and 26 mm), polygalacturonase (PG; 47 and 11 mm), and cellulase (CEL; 22 and 4 mm), except for Vicozyme (V; 65 and 58 mm). The water check isolations (*n* = 4) were negative for both *Leuconostoc* and *Rhizoctonia* in both studies. The *Leuconostoc* checks (*n* = 4) were positive at high concentrations (solid streak of bacteria) of *Leuconostoc* in Study 1 and positive in three of four isolations in Study 2, while negative for *R. solani*. The *R. solani* checks (*n* = 4) were positive for *R. solani* in one of four isolations for both studies. The *R. solani* checks were frequently invaded by bacteria and were positive at low concentrations (a few isolated colonies in bacterial streak) for *Leuconostoc* in three of four isolations for both studies. In enzyme–*Leuconostoc* combination treatments, *Leuconostoc* was found in 14 of 14 and 13 of 14 isolations (at high concentrations) in Test 1 and Test 2, respectively, while *R. solani* was not isolated. In enzyme treatments without *Leuconostoc*, *Leuconostoc* was found in 6 of 14 isolations in both studies at low concentrations, while *R. solani* was not isolated.

### 2.2. Expression of R. solani Pectin Lyase (PNL), Polygalacturonase (PG), and Cellulase (CEL) Genes Were Highly Upregulated during Early Root Infection Stages

**Figure 2:**
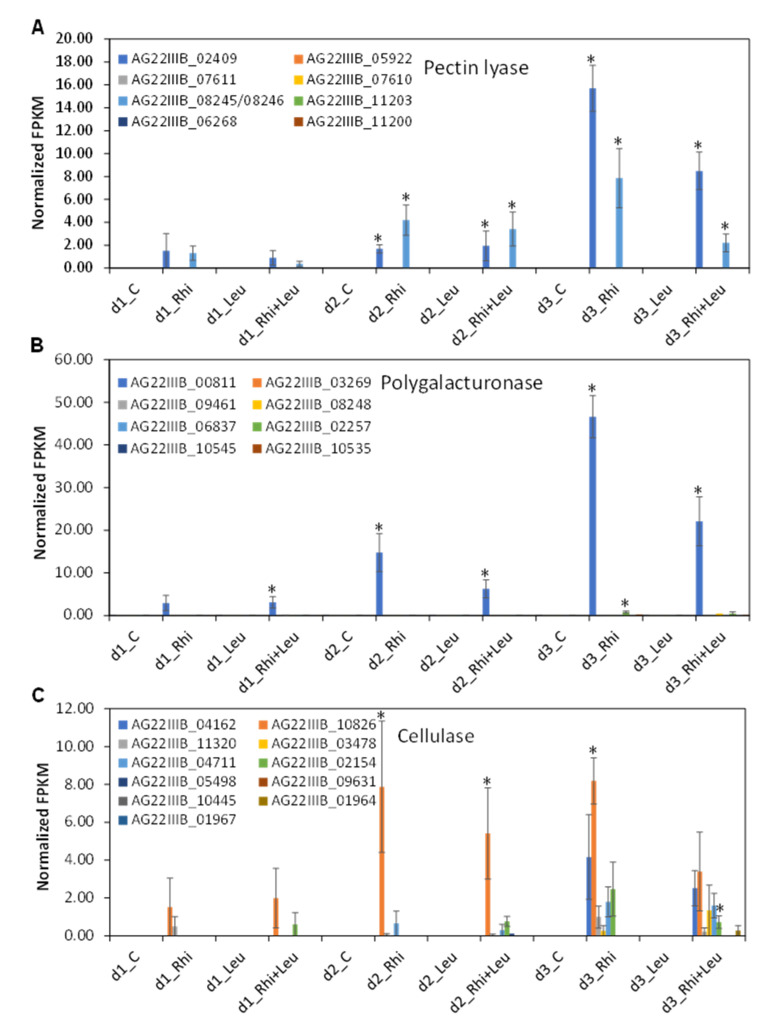
Specific members of *Rhizoctonia solani* (Rhi)-derived plant cell-wall-degrading gene families are highly upregulated during early stages of sugar beet root infection during its interaction with *Leuconostoc mesenteroides* (Leu) or sole infection. (**A**) Expression of pectin lyase; (**B**) polygalacturonase; and (**C**) cellulase gene families at 1, 2, and 3 dpi. Data are mean ± SE of four biological replicates; * *p* ≤ 0.05 between mock control (**C**) and treatments.

As root rot symptoms were increased by the exogenous application of PCWDEs, especially PNL, PG, and CEL when combined with the pathogens, we therefore looked at the expression of *R. solani PNL*, *PG*, and *CEL* genes during root infection in the presence or absence of *L. mesenteroides*. Expression values of these genes at different time points and treatments were extrapolated from the RNAseq data. Gene expression patterns were investigated to understand their roles in early root pathogenesis besides comparing their expressions to the exogenous enzyme application and root rot symptoms. The two expressed *PNL* genes at all time timepoints include *AG2-2IIIB_02409* and *AG2-2IIIB_08245/08246* among eight to nine paralogues (Figure 2A). Their expression increased over time and *AG2-2IIIB_02409* showed the highest expression (Fragments Per Kilobase of transcript per Million mapped reads; FPKM: 15.7) at 3 dpi. Among the three *R. solani* gene families that code for PCWDEs and were used in this study, *PG* genes showed the highest expression at early stages of sugar beet root infection by *R. solani* (Figure 2B). Out of eight paralogues of *R. solani PG* genes, expression of *AG2-2IIIB_00811* was detected at the earliest timepoint (1 dpi) and was highest at 3 dpi (FPKM: 46.58) in comparison to the other paralogues, namely *CEL* and *PNL* genes. Expression of *AG2-2IIIB_00811* increased by >4.5-fold and >15-fold at 2 dpi and 3 dpi respectively in comparison to its expression at 1 dpi. The other expressed *PG* gene paralogues include *AG2-2IIIB_02257* and *AG2-2IIIB_10535*, but their expression was very low (FPKM: 0.09–0.79) and mainly expressed at 3 dpi. Among the 11 *CEL* genes identified in our study, the expressed paralogues were *AG2-2IIIB_04162*, *AG2-2IIIB_10826*, *AG2-2IIIB_11320*, *AG2-2IIIB_03478*, *AG2-2IIIB_04711*, and *AG2-2IIIB_02154* (Figure 2C). In general, their expression was low to moderate. Expression of *AG22IIIB_10826* was detected at 1 dpi, and that increased over time and was highest at 3 dpi (FPKM: 8.18). In general, expression of *R. solani PG*, *PNL*, and *CEL* genes were lower in *R. solani* + *L. mesenteroides*-infected sugar beet roots in comparison to *R. solani*-infected roots only.

### 2.3. Carbohydrates Were Greatly Altered in the Infected Sugar Beet Roots

**Figure 3:**
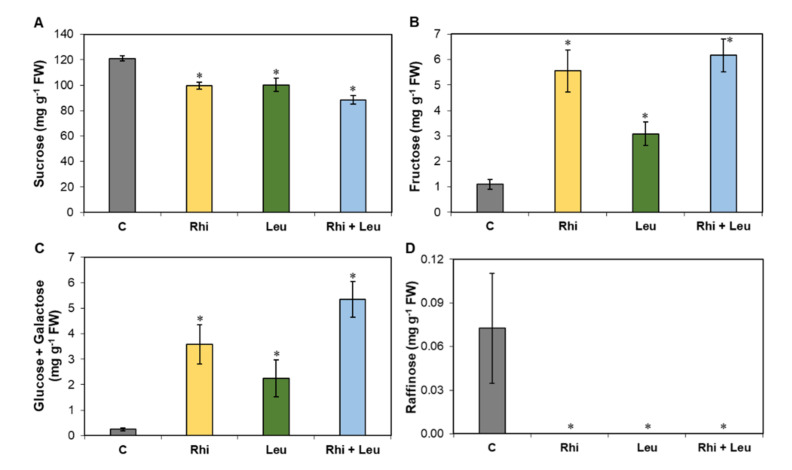
Carbohydrates are highly altered in the *Rhizoctonia solani* (Rhi) or *Leuconostoc mesenteroides* (Leu)-infected sugar beet roots at 3 days post-infection (dpi) vs. mock control (C). Cellular contents of: (**A**) glucose + galactose; (**B**) fructose; (**C**) sucrose; and (**D**) raffinose. The data are mean ± SE of six biological replicates; * *p* < 0.05 between mock control (**C**) and treatments.

At 3 dpi, the cellular content of sucrose decreased (vs. control) by 18% in the *R. solani* and *L. mesenteroides*-infected roots and showed the highest decrease (27%) when both pathogens infected the roots together (Figure 3A). Fructose content increased by 500% and 280% when sugar beet roots were infected by *R. solani* and *L. mesenteroides*, respectively, in comparison to the control samples (Figure 3B). This increase was even higher (~560%) when the pathogens co-infected. The cellular content of glucose + galactose increased by 1440% and 900% in the *R. solani* and *L. mesenteroides*-infected roots, respectively (Figure 3C), and co-infection resulted in the highest increase (2146%). Raffinose was present in minute amounts in the control samples only (Figure 3D).

### 2.4. Percentage of Root N Was Highly Increased upon Infection

**Figure 4:**
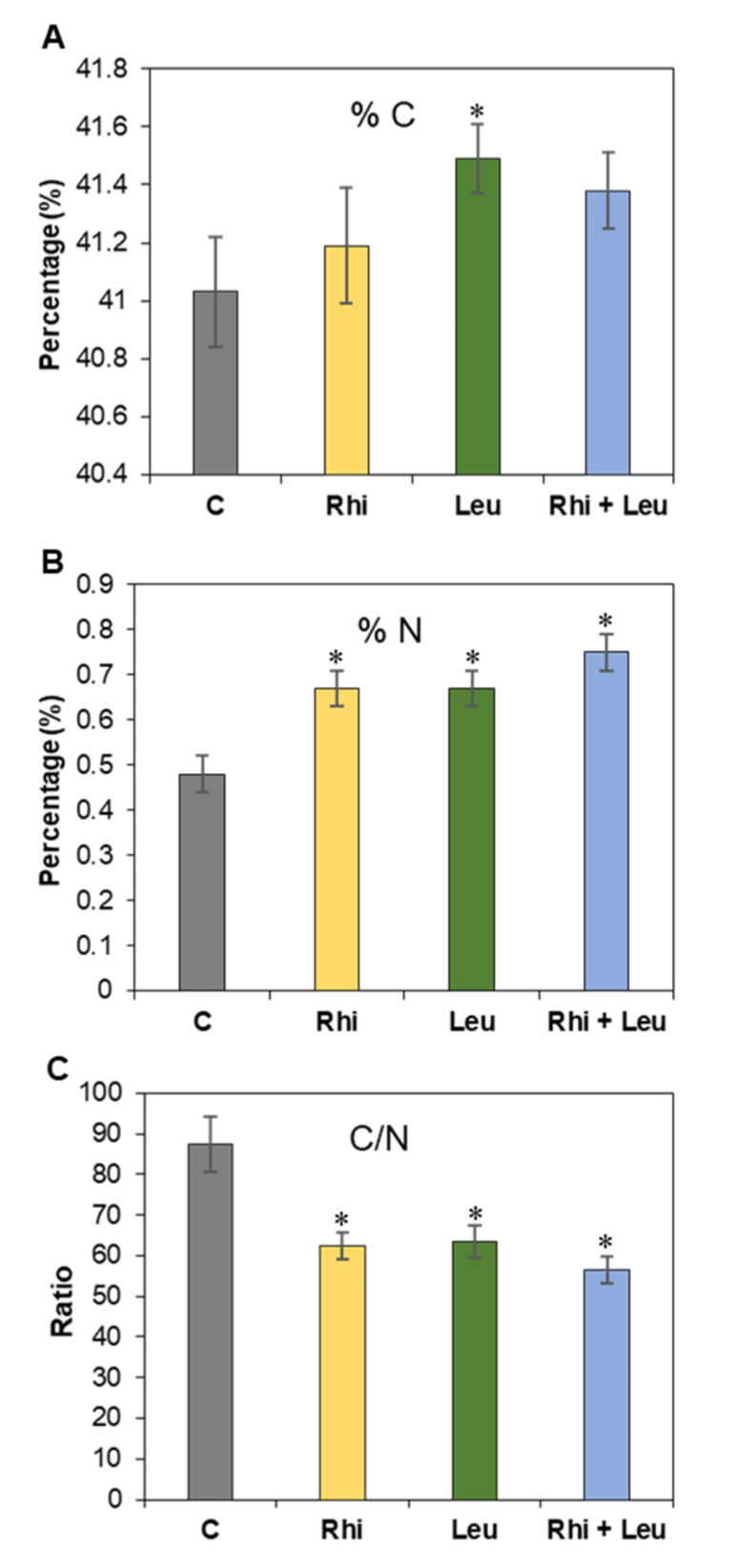
Nitrogen content is highly altered in the *Rhizoctonia solani* (Rhi) or *Leuconostoc mesenteroides* (Leu)-infected sugar beet roots at 3 dpi vs. mock control (**C**). (**A**) % Carbon (C); (**B**) % nitrogen (N); and (**C**) C/N ratio. The data are mean ± SE of six biological replicates; * *p* < 0.05 between mock control (**C**) and treatments.

Percentage of N in the roots was highly altered depending upon the treatment type (Figure 4). Sugar beet roots treated with *R. solani* and/or *L. mesenteroides* did not show any change in C content except for *L. mesenteroides*-infected roots, which showed a small increase in C in comparison to the mock samples at 3 dpi (Figure 4A). Nitrogen content increased (vs. control) by 40% in the *R. solani* and *L. mesenteroides*-infected roots, and this increase was even higher (56%) when roots were infected by both pathogens (Figure 4A). The ratio of C/N decreased by ~29% in the *R. solani* and *L. mesenteroides*-treated roots, and this decrease was higher (36%) when the two pathogens co-infected (Figure 4C).

### 2.5. Differentially Expressed Genes at Early Infection Stages in Sugar Beet, R. solani, and L. mesenteroides in Response to the Treatments

**Figure 5:**
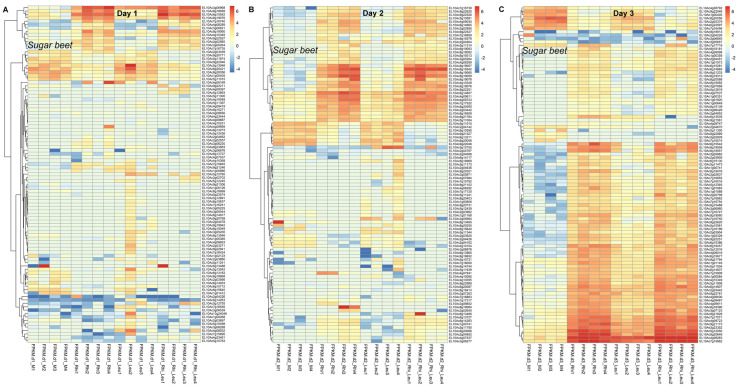
Sugar beet genes are highly induced in the roots exposed to *Rhizoctonia solani* (Rhi) and/or *Leuconostoc mesenteroides* (Leu). Heatmap of log-transformed FPKM values of differentially expressed sugar beet genes at: (**A**) 1 dpi, (**B**) 2 dpi, and (**C**) 3 dpi; *p* ≤ 0.05 between mock control (M) and treatments.

We performed a global RNAseq analysis of all three living systems (sugar beet, *R. solani*, *L. mesenteroides*) to understand the changes in gene expression during their interactions at the early infection stages that resulted in the development of disease symptoms at later stages. Genes that showed major changes in expression in sugar beet, *R. solani*, and *L. mesenteroides* are described here. At 1 dpi, the sugar beet genes that were upregulated (>29-fold) in the presence of only *R. solani* were *peroxidase 27* (*EL10Ac6g15542*) and *auxin-binding protein ABP19b* (*EL10Ac8g19059*) (Table 2; Figure 5A). Examples of genes that were highly upregulated (~2 to 7-fold) by *L. mesenteroides* only include the putative *lipid-binding protein AIR1B* (*EL10Ac5g13046*) and the *auxin-repressed 12.5 kDa protein isoform X1* (*EL10Ac8g20421*), etc., whereas genes that were highly upregulated (up to 348-fold) by both *R. solani* and *L. mesenteroides* include *polygalacturonase inhibitor 1* (*EL10Ac3g06968*) and the *auxin-binding protein ABP19b-like* (*EL10Ac8g19076*), etc. Downregulated genes (up to 21-fold) in the presence of both pathogens and more so by *R. solani*, include *aquaporin TIP2-1* (*EL10Ac9g22046*), and probably *xyloglucan endotransglucosylase/hydrolase protein 6* (*EL10Ac5g11874*), etc. At 2 dpi, sugar beet genes that were highly upregulated (~25 to 300-fold), mainly by *R. solani*, were *peroxidase 27* (*EL10Ac6g15542*) and *E3 ubiquitin-protein ligase ATL31* (*EL10Ac6g14807*), etc., (Table S1; Figure 5B). Examples of sugar beet genes that were upregulated (~6 to 39-fold) by *L. mesenteroides* only include mitochondrial import inner membrane *translocase subunit TIM8* (*EL10Ac5g11344*) and *V-type proton ATPase subunit C* (*EL10Ac3g05664*), etc., whereas genes that were upregulated (~34 to 178-fold) in the presence of both pathogens were *auxin-binding protein ABP19b* (*EL10Ac8g19059*) and *4,5-DOPA dioxygenase extradiol* (*EL10Ac4g09723*), etc. Genes that were downregulated (>7-fold) mainly by *R. solani*, and to some extent by *L. mesenteroides*, were *gibberellin-regulated protein 6* (*EL10Ac5g11015*) and *ethylene-responsive transcription factor ERF003* (*EL10Ac2g04142*), etc. At 3 dpi, an example of a gene that was upregulated (~2-fold) mainly by *R. solani* infection (Table S2; Figure 5C) was *alcohol dehydrogenase class-3* (*EL10Ac7g17719*). Genes that were upregulated (>63-fold) by both pathogens but showed greater-fold change in the presence of *R. solani* were *peroxidase 4* (*EL10Ac7g15862*) and *pathogenesis-related protein PR-4* (*EL10Ac4g08263*), etc., whereas downregulated (>39-fold) genes in the presence of both pathogens, but showing greater-fold change in the presence of *R. solani*, include *gibberellin-regulated protein 14* (*EL10Ac2g03379*) and *peroxidase 42* (*EL10Ac8g20056*), etc.

**Figure 6:**
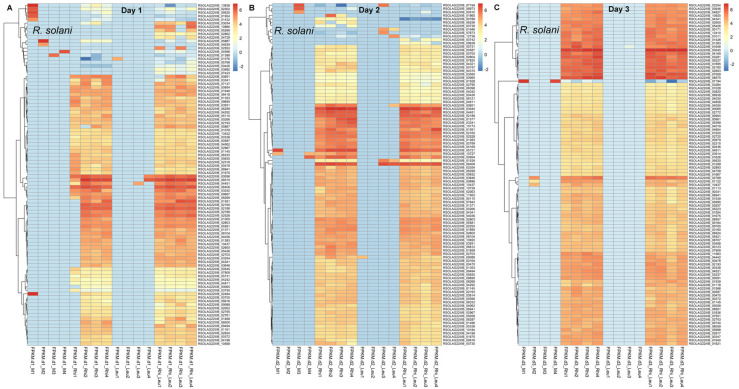
*Rhizoctonia solani* (Rhi) genes are highly induced in the sugar beet roots upon interaction with sugar beet alone or in combination with *Leuconostoc mesenteroides* (Leu). Heatmap of differentially expressed *R. solani* genes at: (**A**) 1 dpi, (**B**) 2 dpi, and (**C**) 3 dpi; *p* ≤ 0.05 between mock control (M) and treatments.

At 1 dpi, *R. solani* genes that were highly expressed include *ADP, ATP carrier protein* (*RSOLAG2-2IIIB_02532*), *60S ribosomal protein L33-B* (*RSOLAG2-2IIIB_06515*), elongation factor 1-beta (*RSOLAG2-2IIIB_06408*), *ribosomal protein* (*RSOLAG2-2IIIB_00056, RSOLAG2-2IIIB_02188, RSOLAG2-2IIIB_02160,* and *RSOLAG2-2IIIB_02528*), *glutamine synthetase* (*RSOLAG2-2IIIB_03242*), and *uracil permease* (*RSOLAG2-2IIIB_00651*), etc., (Table 3; Figure 6A). Genes whose expressions increased (>1.2-fold) in the presence of *L. mesenteroides* include *aspartate aminotransferase* (mitochondrial; *RSOLAG2-2IIIB_04036*), *1,4-alpha-glucan-branching enzyme* (*RSOLAG2-2IIIB_01383*), and ribosomal (*RSOLAG2-2IIIB_02553*), etc., and other uncharacterized genes (*RSOLAG2-2IIIB_05266, RSOLAG2-2IIIB_10866*) which expressed only in the presence of *L. mesenteroides*. At 2 dpi, highly expressed *R. solani* genes include *40S ribosomal protein* (*RSOLAG2-2IIIB_03040, RSOLAG2-2IIIB_02160*, and *RSOLAG2-2IIIB_02528*), elongation factor 1-beta (*RSOLAG2-2IIIB_06408*), *D-arabinitol dehydrogenase 1* (*RSOLAG2-2IIIB_04451*), *peptidyl-Lys metalloendopeptidase* (*RSOLAG2-2IIIB_01577*), *deuterolysin M35 metalloprotease* (*RSOLAG2-2IIIB_03341*), several hypothetical/uncharacterized proteins (*RSOLAG2-2IIIB_05721, RSOLAG2-2IIIB_00954*), *ribosomal protein S25* (*RSOLAG2-2IIIB_02188*), *uracil permease* (*RSOLAG2-2IIIB_00651*), *proteinase T-like* (*RSOLAG2-2IIIB_10173*), and *1,4-alpha-glucan-branching enzyme* (*RSOLAG2-2IIIB_01383*), etc., (Table S3; Figure 6B). Examples of genes that were highly downregulated (>1.6-fold) or had no expression in the presence of *L. mesenteroides* include *copper amine oxidase 1* (*RSOLAG2-2IIIB_01858*) and *60S acidic ribosomal protein P0* (*RSOLAG2-2IIIB_01965*), respectively. Some of the highly expressed genes at 3 dpi were 40S/60S ribosomal protein (*RSOLAG2-2IIIB_03040, RSOLAG2-2IIIB_08570, RSOLAG2-2IIIB_02160, RSOLAG2-2IIIB_00227*, and *RSOLAG2-2IIIB_07009*), *cytochrome c1*, *heme protein*, *mitochondrial* (*RSOLAG2-2IIIB_02803*), and *guanine nucleotide-binding protein subunit beta* (*RSOLAG2-2IIIB_04169*), etc., (Table S4; Figure 6C). Examples of upregulated (>1.4-fold) genes in the presence of *L. mesenteroides* include *homocitrate synthase*, mitochondrial (*RSOLAG2-2IIIB_01939*), and *pyruvate decarboxylase* (*RSOLAG2-2IIIB_01118*), etc. Genes that were highly downregulated (>1.7-fold) in the presence of *L. mesenteroides* include *alanine-glyoxylate aminotransferase 1* (*RSOLAG2-2IIIB_11563*), *putative 4-hydroxy-2-oxoglutarate aldolase*, mitochondrial (*RSOLAG2-2IIIB_01586*), and *carboxypeptidase Y homolog A* (*RSOLAG2-2IIIB_05058*), etc.

**Figure 7:**
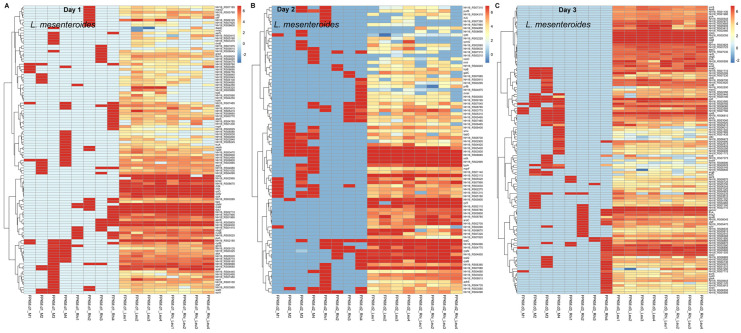
*Leuconostoc mesenteroides* (Leu) genes are highly induced in the sugar beet roots upon interaction with sugar beet alone or in combination with *Rhizoctonia solani* (Rhi). Heatmap of differentially expressed *L. mesenteroides* genes at: (**A**) 1 dpi, (**B**) 2 dpi, and (**C**) 3 dpi; *p* ≤ 0.01 between mock control (M) and treatments.

Genes that were highly expressed (FPKM > 2000) in *L. mesenteroides* and whose expression were even more upregulated in the presence of *R. solani* at 1 dpi include *ATP synthase subunit epsilon* (*NH16_RS02710*), *translation initiation factor IF-1* (*NH16_RS04570*), and *amino acid ABC transporter permease* (*NH16_RS02995*), etc., (Table 4; Figure 7A). Examples of genes that were highly downregulated (>2-fold) in the presence of *R. solani* include amino acid *ABC transporter permease* (*NH16_RS09120*), *D-alanyl-lipoteichoic acid biosynthesis protein dltD* (*NH16_RS02465*), and *nicotinate phosphoribosyltransferase* (*NH16_RS01415*). Some of the highly expressed (FPKM~1400-3000) genes at 2 dpi include translation initiation factor IF-1 (*NH16_RS04570*), *DNA-directed RNA polymerase subunit alpha* (*NH16_RS04590*), and amino acid *ABC transporter permease* (*NH16_RS02995*), etc., (Table S5; Figure 7B). At 3 dpi, highly expressed (FPKM~1200-4000) genes were *50S ribosomal protein L13* (*NH16_RS04635*), *response regulator transcription factor* (*NH16_RS02055*), and *ABC transporter substrate-binding protein* (*NH16_RS05935*), etc., (Table S6; Figure 7C).

### 2.6. GO and KEGG Analyses of Differentially Expressed Genes

Gene ontology (GO) analysis of sugar beet genes whose transcripts were differentially expressed by pathogen infection at 1 dpi were mainly associated with the plasma membrane, ATP binding, kinase activity, protein phosphorylation, and microtubule-related activities to name a few (Figure S1A). At 2 dpi, differentially expressed genes were associated with the plasma membrane, chloroplast, cytosol, the integral component of the membrane, and ATP binding, etc., (Figure S1B). The categories of genes at 3 dpi were like that of 2 dpi, but included other types like protein phosphorylation, serine/threonine kinase, and oxidation-reduction-related, etc., (Figure S1C).

In *R. solani* at 1 dpi, differentially expressed genes were associated with the ribosomal subunit, translation, and the tricarboxylic acid cycle to name a few (Figure S2A). The trend was similar at 2 dpi but included other categories of genes, such as a cellular response to drug-related genes (Figure S2B). At 3 dpi, differentially expressed genes were mainly related to the ribosome, hyphal growth, intracellular protein transport, and vesicle-mediated transport, etc., (Figure S2C).

In *L. mesenteroides* at 1 dpi, differentially expressed genes were primarily associated with penicillin binding (Figure S3A). At 2 dpi, differentially expressed genes included DNA-directed 5′-3′ RNA polymerase activity and the cytosolic small ribosomal subunit (Figure S3B), and at 3 dpi, genes were primarily associated with aldehyde-lyase activity (Figure S3C).

**Figure 8:**
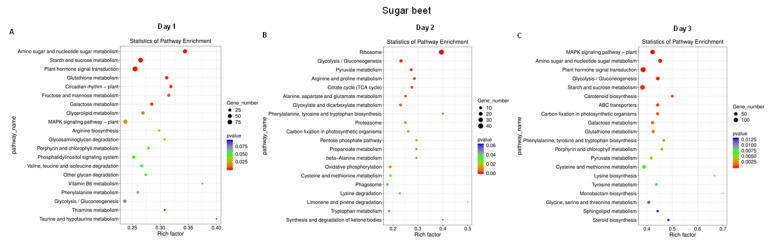
Pathway enrichment of sugar beet genes shows a distinct pattern with infection stages during interaction with *Rhizoctonia solani* and *Leuconostoc mesenteroides*. *Kyoto Encyclopedia of Genes and Genomes* (KEGG) enrichment of sugar beet genes at: (**A**) 1 dpi; (**B**) 2 dpi; and (**C**) 3 dpi. Data are mean of four biological replicates.

Pathway enrichment analysis of sugar beet genes at 1 dpi represented genes mainly associated with amino sugar and nucleotide sugar metabolism, starch and sugar metabolism, plant hormone signal transaction, and glutathione metabolism, etc., (Figure 8A). At 2 dpi, differentially expressed genes were mainly associated with the ribosome, glycolysis/gluconeogenesis, pyruvate metabolism, Arginine (Arg)/Proline (Pro) metabolism, and Alanine (Ala)-Aspartate (Asp)-Glutamate (Glu) metabolism, etc., (Figure 8B). Some of the differentially expressed genes at 3 dpi were associated with the mitogen-activated protein kinase (MAPK) signaling pathway, amino sugar and nucleotide sugar metabolism, plant hormone signal transaction, starch, and sucrose metabolism, etc., (Figure 8C).

**Figure 9:**
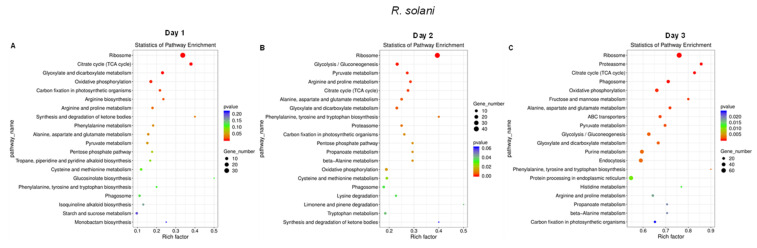
Pathway enrichment of *Rhizoctonia solani* genes shows differential responses with infection stages during interaction with the sugar beet and *Leuconostoc mesenteroides*. *Kyoto Encyclopedia of Genes and Genomes* (KEGG) enrichment of *R. solani* genes at: (**A**) 1 dpi; (**B**) 2 dpi; and (**C**) 3 dpi. Data are mean of four biological replicates.

On the other hand, pathway enrichment analysis of *R. solani* genes that were differentially expressed at 1 dpi were mainly associated with the ribosome, tricarboxylic acid (TCA) cycle, glyoxylate and dicarboxylate metabolism, and oxidative phosphorylation, etc., (Figure 9A). At 2 dpi, differentially expressed genes were mainly associated with the ribosome, glycolysis/gluconeogenesis, pyruvate metabolism, TCA cycle, Arg and Pro metabolism, and Ala/Asp/Glu metabolism (Figure 9B). At 3 dpi, some additional categories of genes associated with the ribosome, proteasome, phagosome, and oxidative phosphorylation, etc., were observed (Figure 9C). Based on the types of genes that were differentially expressed in *L. mesenteroides*, we were unable to perform GO and pathway enrichment analyses.

Pathway enrichment analysis of *L. mesenteroides* differentially expressed genes were primarily associated with the ribosome and selenocompound metabolism at 1 dpi, and RNA polymerase-related at 2 dpi (Figure S4A,B). At 3 dpi, additional categories of genes related to the ascorbate and aldarate, histidine, phenylalanine, tyrosine, and alkaloid (tropane, piperidine, and pyridine) metabolism were highly evident (Figure S4C).

### 2.7. WGCNA Analyses of Differentially Expressed Genes

**Figure 10:**
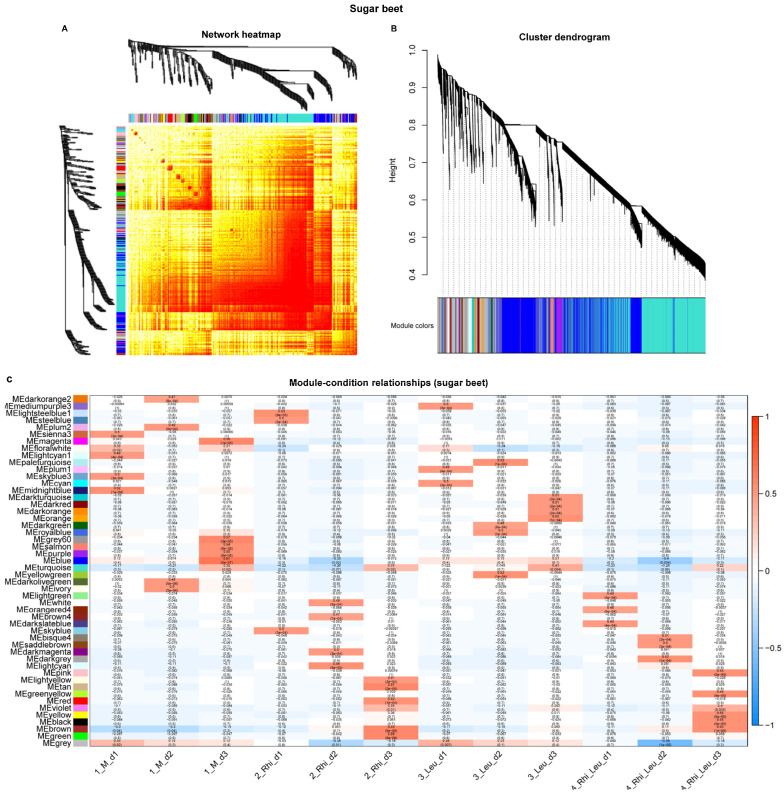
Weighted gene co-expression network analysis (WGCNA) of sugar beet genes infected with or without *Rhizoctonia solani* and *Leuconostoc mesenteroides* showing distinct clustering patterns with infection stages and treatment types. (**A**) Heatmap showing network of differentially expressed genes; (**B**) gene cluster dendrogram; and (**C**) module-condition relationship. Data are mean of four biological replicates. Modules with high correlation values (>0.5) and significance (*p* < 0.05) are boxed with red rectangles.

WGCNA analysis was performed using the count data of RNA-sequencing reads mapped to the genomes of all three organisms used in this study. The WGCNA analysis evaluates the pairwise correlation between genes across samples. The correlation coefficient was determined for all genes and a hierarchical clustering tree was constructed using the correlation matrices. Gene modules are represented by different colors and each module consists of several genes. The heatmap and gene cluster dendrogram of sugar beet genes are shown in Figure 10A,B. Highly co-expressed (positive correlation >0.5; significance of *p* < 0.05) gene modules in sugar beets (Figure 10C) in response to *R. solani* inoculation at 1 dpi were represented by MElightsteelblue1, MEsteelblue, and MEskyblue. At 2 dpi, gene modules detected were MEwhite, MEbrown4, MEdarkmagenta, and MElightcyan, and at 3 dpi, detected modules were MElightyellow, MEtan, MEred, MEviolet, MEbrown, and MEgreen. Sugar beet *L. mesenteroides*-responsive gene modules at 1 dpi were MEmediumpurple3, MEplum1, and MEcyan. At 2 dpi, they were MEpaleturquoise, MEdarkgreen, MEroyalblue, and MEyellowgreen, and at 3 dpi, they were MEdarkturquoise, MEdarkred, MEdarkorange, and MEorange. Sugar beet gene modules responsive to *R. solani* + *L. mesenteroides* at 1 dpi were represented by MElightgreen, MEorangered4, and MEdarkslateblue. At 2 dpi, the detected modules were MEbisque4, MEsaddlebrown, and MEdarkgrey, and at 3 dpi, they were MEpink, MEgreenyellow, MEviolet, MEyellow, MEblack, and MEbrown.

**Figure 11:**
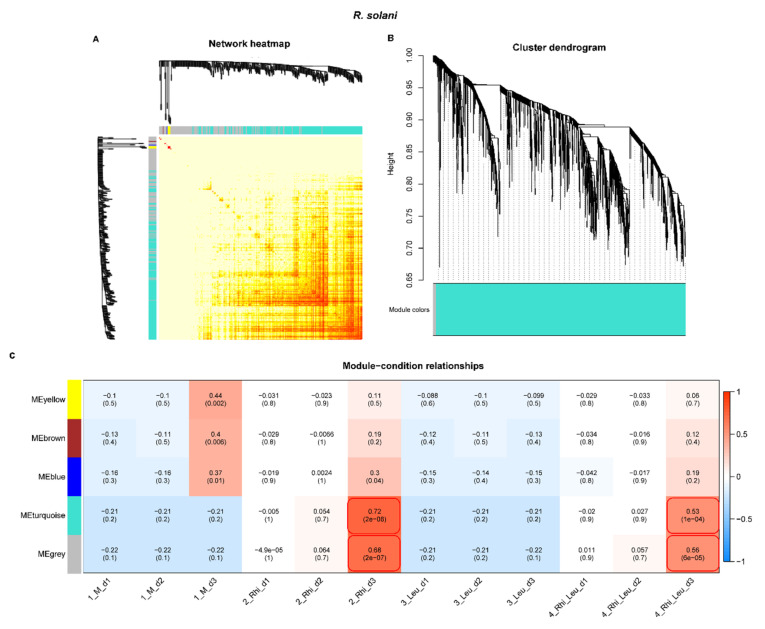
Weighted gene co-expression network analysis (WGCNA) of *Rhizoctonia solani* genes during interactions with the sugar beet in the presence or absence of *Leuconostoc mesenteroides* shows distinct clustering patterns with infection stages and treatment types. (**A**) Heatmap showing network of differentially expressed genes; (**B**) gene cluster dendrogram; and (**C**) module-condition relationship. Data are mean of four biological replicates. Modules with high correlation values (>0.5) and significance (*p* < 0.05) are boxed with red rectangles.

The heatmap and gene cluster dendrogram of *R. solani* genes are shown in Figure 11A,B. The highly co-expressed (positive correlation > 0.5; significance of *p* < 0.05) *R. solani* gene modules for the *R. solani* only inoculation of sugar beets were represented by MEturquoise, MEgrey, and MEblue, and for the *R. solani + L. mesenteroides* inoculation they were represented by MEturquoise and MEgrey at 3 dpi only (Figure 11C).

The heatmap and gene cluster dendrogram of *L. mesenteroides* genes are shown in Figure S5A,B. The highly co-expressed (positive correlation > 0.5; significance of *p* < 0.05) *L. mesenteroides* gene module at 3 dpi, while infecting sugar beets alone or with *R. solani + L. mesenteroides* inoculation, was MEturquoise (Figure S5C), whereas at 2 dpi, the *L. mesenteroides* gene module for the *R. solani + L. mesenteroides* inoculation was represented by MEsalmon (Figure S5C).

## 3. Discussion

### 3.1. R. solani-Derived Plant Cell-Wall-Degrading Enzymes Play a Key Role in Increasing Root Rot

Plant cell-wall-degrading enzymes in phytopathogenic fungi are well known for their critical role in plant pathogenesis [14]. They not only depolymerize complex cell wall polysaccharides, thereby facilitating fungal entry inside the plant cell, but the simpler sugars derived from cell wall degradation can also serve as an easy source of energy during early pathogenesis. Among the different PCWDEs produced by *R. solani*, pectin-digesting enzymes play a critical role in successful pathogenesis and disease development [15,16,17]. The enzyme PG hydrolyzes pectate by cleaving the α-(1,4)-glycosidic bonds. The other PCWDE used in this study, CEL, targets cellulose present in the cell wall and catalyzes the degradation of cellulose by targeting the *β*-1,4-glycosidic bonds. Cellulase isolated from *R. solani* has been shown to act as an elicitor during plant–pathogen interactions, though its enzyme activity is not required for such elicitor activity [18]. Earlier studies involving PCWDE-related genes have demonstrated the role of these enzymes in fungal pathogenicity, including *R. solani* [15,16,17]. The goal of this study was to delineate precisely the role of *R. solani* PCWDEs, namely PNL, PG, and CEL, in increasing sugar beet root rot symptoms when in close contact with *L. mesenteroides* in the soil. We used both exogenously supplied purified enzymes in combination with *L. mesenteroides* or *L. mesenteroides* alone, and *R. solani + L. mesenteroides* inoculations without any PCWDEs. The data presented here suggest that pectin-degrading enzymes such as PNL and PG had more measurable root rot than CEL, with PNL showing the most rot among the three enzymes when individual enzymes were combined with *L. mesenteroides* inoculation (Table 1; Figure 1). Other enzymes such as xylanase, pectate lyase, and pectin methylesterase were investigated in preliminary studies, but led to less rot when in combination with *L. mesenteroides* than PNL, PG, and CEL (data not shown). The highest root rot in the case of ‘V’ was not surprising as this is an admixture of multiple PCWDEs. The enzyme data was further supported by the expression of *R. solani PNL*, *PG*, and *CEL* genes that were highly induced at early stages of root infection by *R. solani* alone or during co-infection with *L. mesenteroides* (Figure 2). Though a greater number of *CEL* genes were expressed in comparison to *PNL* and *PG* genes, the overall expression of *PNL* and *PG* was higher than *CEL*, and were highest at 3 dpi.

### 3.2. Altered Cell Wall Metabolism Affects Carbohydrate Contents and Decreases C/N Ratio upon Infection

An increase in simpler carbohydrates, namely fructose and glucose + galactose upon *R. solani* and *L. mesenteroides* infections and more so when the pathogens co-infected sugar beet roots (Figure 3), suggest a breakdown of complex cell wall carbohydrates into simpler carbohydrates, thereby promoting pathogenesis [19]. On the other hand, decreases in sucrose content (18%) upon infections by individual pathogens, and even more (27%) during co-infection (Figure 3A), suggest that the rapid utilization of stored sucrose in roots was an easy source of C by the invading pathogens. Studies on pathogen infections of sugar beet roots during storage, have reported similar findings where sucrose content decreased, and glucose and fructose increased [20]. We also investigated if changes in carbohydrate content during pathogenesis affected total C and N contents in the roots. Total C was primarily unaltered except for the *L. mesenteroides*-infected roots (Figure 4A). An increase in total N in the infected roots (Figure 4B) indicated that there was potentially a high demand for N during fungal and bacterial pathogenesis. This finding was further supported by an increased expression of N metabolism-related genes, both in the roots as well as in *R. solani* (Figure 8 and Figure 9). As N serves as a building block for amino acids and numerous other nitrogenous compounds, there is a high demand for N, both from the host defense perspective as well as the pathogenesis perspective (reviewed in study [21]). Though N metabolism plays a key role in host plant defense against pathogens, there may be a threshold beyond which additional N availability in plants might contribute to host susceptibility. Other factors, such as the form of N supplied, pathogen type (i.e., fungi (biotroph/necrotrophy) or bacteria), or interacting plant species, etc., can contribute to host plant susceptibility or resistance. From the pathogen perspective, there is a high demand for N at infection sites to maintain successful pathogenesis (reviewed in study [21]). During plant–fungal pathogenic interactions, the majority of the N required by fungi is acquired from host plants in the form of amino acids such as GABA, Gln, and Glu, etc., which are present in higher concentrations (millimolar quantities) in comparison to other amino acids in plant cells [22]. Thus, N starvation can regulate pathogenicity-related genes and thereby control disease development [23]. An increase in N content in the infected roots, and a significantly lower C/N ratio (vs. mock; Figure 4C), may indicate an increase in the N-driven primary metabolism as opposed to the C-driven secondary metabolism, such as the biosynthesis of lignin and cellulose, and their roles in reinforcing physical barriers (reviewed in study [24]). The lower C/N ratio in the infected roots, and increased root damage, implicated an increased host susceptibility upon an altered C/N ratio.

### 3.3. Global Gene Expression Analyses Show Distinct Patterns in Sugar Beet, R. solani, and L. mesenteroides during Complex Interactions

A systems biology approach offers tremendous potential to understand complex biological interactions and identify critical components when multiple organisms interact with each other. In our case, sugar beets growing in the field were root inoculated with *R. solani* and/or *L. mesenteroides* and we evaluated global changes in gene expression, both in the sugar beet host and pathogens (*R. solani* and *L. mesenteroides*) during early infection stages ranging between 24 hpi and 72 hpi. Our goal was to understand the changes in early gene expression in sugar beets, and the pathogens (fungal and bacterial) that are associated with host response and pathogenesis respectively.

The gene expression data provide insights on pathogen-specific sugar beet responses, including overlaps between fungal and bacterial pathogens in this study. Some of the highly expressed sugar beet genes due to *R. solani* infection at the earliest time point (1 dpi) included *peroxidase 27* (*EL10Ac6g15542*) and *auxin-binding protein ABP19b* (*EL10Ac8g19059*), etc. The higher expression of peroxidase and phytohormone-related genes (such as auxin, gibberellin) observed here possibly indicates their role in host defense at early infection stages (Table 2; Figure 5A). The similar role of some of these genes against other *R. solani* strains have been reported in diverse plant species, including soybean [25], potato [26], rice [27], and sugar beet [28,29]. Sugar beet genes were highly upregulated (up to 7-fold) by *L. mesenteroides* such as *lipid-binding protein AIR1B* (*EL10Ac5g13046*) and *auxin-repressed 12.5 kDa protein isoform X1* (*EL10Ac8g20421*), which show bacterial pathogen-specific sugar beet responses (Table 2), whereas other highly upregulated (up to 348-fold) sugar beet genes, such as *polygalacturonase inhibitor 1* (*EL10Ac3g06968*) and *auxin-binding protein ABP19b-like* (*EL10Ac8g19076*) highlight the global response of the sugar beet against fungal and bacterial pathogens (Table 2). An increase in the expression of the sugar beet *polygalacturonase inhibitor* gene (*EL10Ac3g06968*) corroborates well with the observation from the exogenous enzyme application study, whereby the PG enzyme had a significant effect on increasing root damage. An increase in *R. solani PG* genes during early infection stages reiterates the fact that this gene family is highly critical for successful pathogenesis. The future RNAi-mediated targeting of specific members of the *R. solani PG* gene family, along with other candidates, may be highly effective in improving sugar beet host resistance against both *R. solani* and *L. mesenteroides*. Furthermore, designing inhibitors specific to PG, or other PCWDEs such as PNL, may serve as an alternative approach to improve host resistance. Further insights into the candidate genes that were highly co-expressed in the gene modules at the earliest infection stage (1 dpi) revealed a unique sugar beet gene expression against *R. solani* (ME_lightsteelblue1: *heavy-metal-associated* (*EL10Ac3g05414*), *methionine-tRNA ligase* (*EL10Ac8g19256*), *ATHILA ORF-1 family* (*EL10Ac4g08425*), and *U-box domain-containing protein 43* (*EL10Ac1g01912*), etc.), *L. mesenteroides* (ME_mediumpurple3: *proliferating cell nuclear antigen 2* (*EL10Ac1g00531*), *FAS1 domain-containing protein* (*EL10Ac5g10640*), and *retrovirus-related Pol polyprotein* (*EL10Ac5g11625*), etc.), and *R. solani* + *L. mesenteroides* (ME_lightgreen: *E3 ubiquitin-protein ligase* (*EL10Ac1g01117*), *mechanosensitive ion channel protein 10* (*EL10Ac4g10289*), and *organic cation/carnitine transporter 2* (*EL10Ac9g22914*), etc.). Sugar beet genes that were downregulated by both pathogens include *aquaporin TIP2-1* (*EL10Ac9g22046*) and probably *xyloglucan endotransglucosylase/hydrolase* (*XTH*) *protein 6* (*EL10Ac5g11874*), etc. Both genes have been demonstrated to confer host resistance against pathogens in plants [30,31]. The role of aquaporins in host plant resistance against diverse types of pathogens have been reported in several studies [31,32]. In citrus (*Citrus sinensis*), the *CsXTH04* gene was highly upregulated in the citrus bacterial canker-resistant varieties and were induced by phytohormones namely salicylic acid and methyl jasmonate [30]. Downregulation of both genes, during susceptible interaction in our case and evidence from past reports taken together into consideration, might suggest the potential role of these genes in sugar beet host resistance. The future overexpression of these candidates, and an evaluation for resistance, will demonstrate their precise roles in resistance against *R. solani* and other pathogens.

Gene expression in *R. solani* showed differential responses during pathogenic interaction with the sugar beet or combined interaction with the sugar beet and *L. mesenteroides*. The role of *R. solani* PCWDEs in fungal pathogenesis is well documented [14] and was discussed earlier in the context of the interaction with *L. mesenteroides* during sugar beet root infection. Besides PCWDEs, some of the other highly upregulated *R. solani* genes at the earliest time point (1 dpi), both during interaction with sugar beet roots singly or in combination with *L. mesenteroides*, include *ADP*, *ATP carrier protein* (*RSOLAG2*-*2IIIB_02532*), *40S ribosomal protein* (*RSOLAG2-2IIIB_00056*, *RSOLAG2-2IIIB_02160*, *RSOLAG2-2IIIB_02528*), *60S ribosomal protein L33-B* (*RSOLAG2-2IIIB_06515*), *glutamine synthetase-like* (*RSOLAG2-2IIIB_03242*), and *uracil permease* (*RSOLAG2-2IIIB_00651*), etc., (Table 3; Figure 6A). Some of them have been functionally characterized in other fungi, demonstrating their critical role in plant pathogenesis. As an example, the mutation of the *ADP, ATP carrier protein* (transfers ATP from mitochondria to cytoplasm) gene in *Verticillium dahliae* significantly reduced the fungal pathogenicity of the mutant strain and decreased disease symptoms in tobacco [33]. The highest expression of the *ADP*, *ATP carrier protein* gene (*RSOLAG2*-*2IIIB_02532*) in our case at 1 dpi will be an exciting candidate for further investigation to determine its role as a pathogenicity factor, functionally validating other uncharacterized genes that were highly expressed at this time point. Some of the highly co-expressed *R. solani* candidate genes identified at 1 dpi belonged to the module ME_turquoise, including *2-oxoglutarate dehydrogenase, mitochondrial* (*RSOLAG2-2IIIB_08262*), *clathrin heavy chain* (*RSOLAG2-2IIIB_02720*), endoglucanase 5 (*RSOLAG2-2IIIB_02295*), and *E3 ubiquitin-protein ligase HUWE1* (*RSOLAG2-2IIIB_00065*), etc. A few of these genes have been demonstrated to be important pathogenicity factors in other fungi. A T-DNA insertional mutation of the clathrin heavy chain (*CHC*) gene in *Botrytis cinerea* showed a significant reduction in fungal *pathogenicity* during the infection of the bean leaf, cucumber cotyledon, and apple fruit [34]. In another study, the inhibition of serine palmitoyltransferase activity using chemical inhibitors showed antifungal activity against *Aspergillus* *fumigates* and *Rhizopus oryzae*, *Candida* sp. [35]. Some of the highly expressed *R. solani* candidate genes such as *RSOLAG2*-*2IIIB_02532*, *RSOLAG2-2IIIB_02720* identified here could be potential targets for future RNAi implementation in sugar beets to improve host resistance. *Rhizoctonia solani* genes that were further upregulated (such as *RSOLAG2-2IIIB_04036* and *RSOLAG2-2IIIB_01383*) or downregulated (such as *RSOLAG2-2IIIB_01858* and *RSOLAG2-2IIIB_01965*) in the presence of *L. mesenteroides* highlight the complex regulation of gene expression during the fungal–bacterial interaction and could be due to low pH in the infected roots resulting from the rapid multiplication of *L. mesenteroides* [12].

Highly expressed *L. mesenteroides* genes, such as *ATP synthase subunit epsilon* (*NH16_RS02710*), *translation initiation factor IF-1* (*NH16_RS04570*), *amino acid ABC transporter permease* (*NH16_RS02995*), and *DNA-directed RNA polymerase subunit alpha* (*NH16_RS04590*) (Table 4 and Table S5), suggest the importance of these genes in pathogenesis during the early sugar beet root infection stages (Figure 7). Further analysis of candidate genes belonging to the highly co-expressed gene modules is needed, such as those of ME_turquoise, which includes *NH16_RS05440* (*ADP-ribose pyrophosphatase*), *NH16_RS06070* (*prolyl-dipeptidyl aminopeptidase*), *NH16_RS03690* (*glycosyl transferase*), *msrA* (protein repair), and *mgtE* (magnesium transporter), etc. Many of these genes mentioned above have been demonstrated to be important bacterial pathogenicity-related factors and could serve as potential targets for future mitigation strategies [36,37,38]. Besides the genes mentioned above, the high abundance of specific *L. mesenteroides* rRNA genes indicate the role of these genes during early bacterial pathogenesis [39] when rapid multiplication is one of the key events.

Pathway enrichment analysis revealed different strategies employed by sugar beets and *R. solani*. Genes associated with pathways primarily related to carbohydrates, amino acids, amino sugars, cell membranes, and phytohormones played important roles in sugar beet response (Figure 8). However, in *R. solani*, at 1 dpi the metabolic pathways were primarily associated with the ribosome, TCA cycle, and oxidative phosphorylation, etc., and at later stages (3 dpi), pathways related to the ribosome, proteasome, and phagosome, etc., played critical roles in pathogenesis (Figure 9). In *L. mesenteroides*, pathways related to ribosome, selenocompound metabolism and RNA polymerase were predominant during early infection stages (1 and 2 dpi; Figure S4A,B). These data presented here suggest the common role of primary metabolism in all host-plant fungal and bacterial pathogens, besides the role of unique pathways in the pathogens towards maintaining successful pathogenesis and the development of disease symptoms.

## 4. Materials and Methods

### 4.1. Experimental Design, Field Inoculation, and Sample Collection

Two separate studies were conducted in 2020 with 12 (Table 1) of the original 48 treatments that were used in preliminary studies involving *R. solani* and *L. mesenteroides*. The field studies were conducted at the USDA-ARS North Farm (Study 1; 42°33.194′ N, 114°21.490′ W, elevation 1189 m; Study 2, 42°33.175′ N, 114°21.492′ W, elevation 1190 m) in a field with Port Neuf silt loam soil located near Kimberly, ID. The experiments were each arranged in a randomized complete block design (RCBD) with 8 replications. The pathogen strains used in these studies were *Leuconostoc mesenteroides* strain L12311 (haplotype 11) [8] and *R. solani* strain F521 anastomosis group 2-2 IIIB (phylogenetic group PG2; 2). The sugar beet cultivar B-7 (Betaseed Inc.; Kimberly, ID) roots were inoculated on the shoulder of the root (where root meets the soil line) using a cork borer to create a 10 mm diameter × 24 mm deep hole. For the non-inoculated water control (mock; M), 0.1 mL of sterile well water was inserted. For the *L. mesenteroides* treatment, 0.1 mL of a 10^8^ cfu mL^−1^ suspension was inoculated. The *L. mesenteroides* inoculum was prepared using yeast dextrose calcium carbonate agar (YDC), as described previously [3]. For *R. solani* treatment, a 2 × 2 mm piece of mycelial mass was placed in the hole with 0.1 mL of sterile well water. The mycelia were produced by growing the fungus for 10 days in potato dextrose broth (product no. 1.00510.0500; EMD Chemicals Inc., Gibbstown, NJ) using a shaker on the bench top at 22 °C. The mycelia had been rinsed with sterile well water prior to use. For the *L. mesenteroides* + *R. solani* treatment, 0.1 mL of a 10^8^ cfu mL^−1^ suspension was inoculated along with the 2 × 2 mm mycelial mass. The enzymes were inoculated in a total volume of 0.2 mL at the following rates: CEL (156 units), PG (93 units), PNL (107 units), and V (4 units). The same rate was utilized for the enzyme combination treatments. When the enzymes were evaluated in combination with *L. mesenteroides*, the enzymes were diluted in 0.1 mL of a 10^8^ cfu mL^−1^ of bacterial suspension instead of sterile well water. After inoculations, the plug was replaced and sealed with petroleum jelly (Unilever, Greenwich, CT). The fields were planted on 20 April, inoculated on 26 August, and evaluated on 16 October. The roots were evaluated for rot by bisecting the root through the inoculation hole and measuring the amount of rot perpendicular to the plug with a ruler. Representative samples were also photographed. To fulfill Koch’s postulates, 44 isolations per study were conducted from the following treatments (4 isolations from each for the 4 different checks, 14 isolations from treatments with only water, and 14 isolations from treatments with *Leuconostoc* suspension) on fungal and bacterial media, as described previously [12].

For RNAseq work, sugar beet root tissues were collected at 24 h, 48 h, and 72 h post-inoculation from mock and infected samples (tissues from the area of infection), flash frozen in liquid N_2_, and stored at −80 °C until the samples were taken out for grinding in a Geno grinder at an ultralow temperature. Ground materials were stored at −80 °C and subsequently used for RNA extraction and carbohydrate analyses.

### 4.2. Carbohydrate Analysis

Extractions of soluble carbohydrates were performed from approximately 100 mg of finely ground sugar beet root tissues (stored in −80 °C) following the method described here. Soluble carbohydrates were extracted in 1 mL of 80% absolute ethanol at 65 °C for 30 min. Following incubation, the samples were cooled for 5 min, vortexed at a medium speed for 2 min, and centrifuged at 13,000 rpm for 8 min to pellet tissues. The supernatants were filtered using a 0.45 µm nylon syringe filter (Pall Corp., Port Washington, NY, USA). A total of 11 soluble carbohydrates were attempted to quantify using a PerkinElmer series 200 HPLC pump and autosampler (PerkinElmer Inc., Waltham, MA, USA) coupled with a Shimadzu RID-10A refractive index detector set at 30 °C (Shimadzu Scientific Instruments Inc., Columbia, MD, USA). Carbohydrates were separated in a Luna NH2 analytical column (heated to 25 °C, 250 × 4.6 mm, 5 µm, 100 Å; Phenomenex Inc., Torrance, CA, USA) surrounded by an NH2 Guard column (4 × 3 mm, Phenomenex Inc.) using an isocratic mobile phase of 80% acetonitrile at a flow rate of 2 mL min^−1^. Only four carbohydrates that were detected in the sugar beet roots were sucrose, fructose, glucose + galactose, and raffinose. All carbohydrates excluding sucrose, and glucose + galactose, were quantified using a 5-point external standard curve (0.125–2 mg mL^−1^) except for sucrose, where the range of standard curve was from 2 to 18 mg mL^−1^. For quantification of glucose + galactose (the two peaks did not separate), the areas and concentrations of each carbohydrate were added to create a combined standard curve. Analysis of the chromatographs and data processing were performed using PerkinElmer TotalChrom software (version 6.2.1).

### 4.3. Analysis of C and N

Total carbon (C) and nitrogen (N) were measured by high-temperature combustion using a VarioMax CN analyzer (Elementar Americas, Inc. Mt Laurel, NJ, USA) according to the Dumas method [40].

### 4.4. Data Analysis of Enzyme Application and Sugar Beet Root Rot Field Experiments

Normality of the data were assessed using the Univariate procedure, while the homogeneity of variance was determined using Levene′s test in SAS (version 9.4; SAS Institute Inc., Cary, NC, USA). The general linear model procedure (Proc GLM) in SAS was used to conduct the analysis of variance. Mean comparisons were conducted using Fischer′s protected least significant difference (α = 0.05). Mean comparisons across multiple treatments were conducted using single degree-of-freedom orthogonal contrast statements.

### 4.5. RNA Extraction, Construction of mRNA Libraries and Sequencing

Total RNA extraction was performed using the Plant/Fungi Total RNA Purification Kit (Norgen Biotek Corp, ON, Canada) according to the manufacturer’s protocol. The quality and quantity of total RNA were analyzed using Bioanalyzer 2100 (Agilent Technologies, Santa Clara, CA, USA). Acceptable RIN number for samples was >7.0. High-quality RNA was used for the construction of mRNAseq libraries.

Poly(A) RNA sequencing libraries were constructed according to the protocol described in Illumina’s TruSeq-stranded-mRNA sample preparation. Approximately 1 ug of total RNA was used. Ribosomal RNA depletion was performed following the method described in the Ribo-Zero™ rRNA Removal Kit (Illumina, San Diego, CA, USA). Poly(A) mRNAs were purified using oligo-(dT) magnetic beads after two rounds of purification. Poly(A) RNA fragmentation was performed using divalent cation buffer at an elevated temperature. Cleaved RNA fragments were reverse-transcribed to produce cDNAs that were subsequently used to produce U-labeled second-strand DNA. After end repair, 3′ adenylation, adapter ligation, and PCR, final libraries were prepared. Quality control and quantification of the sequencing libraries were performed using Bioanalyzer 2100 (Agilent Technologies, Santa Clara, CA, USA). Illumina’s NovaSeq 6000 sequencing platform at the LC Sciences (Houston, TX, USA) was used for paired-end (150 bp) sequencing following the vendor’s protocol.

### 4.6. Read Mapping and Transcriptome Assembly

Raw reads from mRNA-Seq data were processed to remove any low-quality reads and adapter sequences, using in house (LC Sciences, Houston, TX, USA) Perl scripts and Cutadapt [41]. To analyze sequence quality, FastQC (http://www.bioinformatics.babraham.ac.uk/projects/fastqc/, accessed on 9 April 2021) was performed. Hisat2 [42] software was used to map reads to the sugar beet (*Beta vulgaris* subsp. *vulgaris*) reference genome EL10 (https://phytozome-next.jgi.doe.gov/info/Bvulgaris_EL10_1_0, accessed on 9 April 2021) and StringTie [43] was used to assemble mapped reads originating from each sample. For mRNAseq, we obtained ~44–69 million raw reads/samples and ~41–66 million valid reads/samples for each of the sugar beet, *R. solani*, and *L. mesenteroides* (Tables S7–S9).

### 4.7. Differentially Expressed mRNAs and Bioinformatics Analysis

Transcriptome data originating from different samples were merged using Perl scripts to reconstruct a comprehensive transcriptome data. StringTie [43] and edgeR [44] were used to quantify expression levels of transcripts. Expression level of mRNA expression were calculated by measuring FPKM values using the StringTie software. Differential expression of transcripts was estimated using EdgeR-R packages. Differentially expressed genes (DEGs) were identified using a cutoff *p*-value of < 0.05 and |log2 (fold-change)| > 1.

For annotation of transcripts, Blastx was used against the NCBI database. Gene ontology (GO) analysis was performed by BLAST searching the transcripts to the GO database and calculating gene numbers for each term. Pathway enrichment analysis was performed using the *Kyoto Encyclopedia of Genes and Genomes* (KEGG; [45]).

The weighted gene co-expression network analysis (WGCNA) was performed using the WGCNA package in R (3.2.2.) [46] and according to the methods described in study [47]. The parameters used for this analysis are, minimum module size (minimum module size for module detection)—30; module membership assignment (kME), minCoreKME (a number between 0 and 1. If a detected module does not have at least minCoreKMESize genes with eigengene connectivity, the module is disbanded. Its genes are unlabeled and returned to the pool of genes waiting for module detection)—0.5; min CoreKMESize (see minCoreKME)—minModuleSize/3; minKMEtoStay (genes whose eigengene connectivity to their module eigengene is lower than minKMEtoStay are removed from the module)—0.3. For each block of genes, the network is constructed. Genes are then clustered using average linkage hierarchical clustering and modules are identified in the resulting dendrogram by the Dynamic Hybrid tree cut. Found modules are trimmed of genes whose correlation with module eigengene (KME) is less than minKMEtoStay. Modules in which fewer than minCoreKMESize genes have KME higher than minCoreKME are disbanded.

### 4.8. Data Availability

The raw data resulting from mRNAseq (BioProject ID: PRJNA791627) were submitted to the NCBI SRA database.

## 5. Conclusions

The work presented here shows the precise roles of *R. solani*-derived PCWDEs, especially the PNL, PG, and CEL in sugar beet root rot that significantly increased in the presence *L. mesenteroides*. Amongst the different PCWDEs studied here, PNL had the most effect in increasing root rot. This observation was confirmed by using a combination of approaches including the exogenous application of PCWDEs, quantification of carbohydrates and total C/N, and global gene expression analyses in all three systems during their complex interactions. We have identified potential candidate genes and highly co-expressed gene clusters in sugar beet, *R. solani*, and *L. mesenteroides* that could potentially be critical in host defense and pathogenesis during the early root infection stages under field conditions. Future RNAi-based strategies to target some of these key host and pathogen genes identified in this work have the potential to improve sugar beet host resistance against both *R. solani* and *L. mesenteroides*.

## Figures and Tables

**Table 1:** Rot tests were conducted via two Idaho field studies conducted in 2020 to investigate plant cell-wall-degrading enzymes with and without *Leuconostoc mesenteroides* compared with *L. mesenteroides* and *Rhizoctonia solani* inoculated alone and in combination with the commercial sugar beet cultivar B-7.

Treatment	Enzyme ^y^	Pathogen	Root Rot (mm)
48	V	L12311	65 a
47	V	None	58 ab
12	PNL	L12311	57 b
10	PG	L12311	47 c
4	None	L12311 + F521	33 d
11	PNL	None	26 e
6	CEL	L12311	22 e
3	None	F521	20 e
2	None	L12311	13 f
9	PG	None	11 fg
5	CEL	None	4 gh
1	None	None	3 h
*p* > *F* ^z^			<0.0001
LSD (α = 0.05)			7

^y^ CEL = cellulase from *Aspergillus niger* (Sigma product C1184); None = no enzyme; PG = polygalacturonase from *Rhizopus* (P2401); PNL = pectin lyase from *Aspergillus* (P3026); and V = Vicozyme L, a commercial multienzyme complex (arabanase, cellulase, B-glucanase, hemicellulase, and xylanase; V2010). The enzyme concentrations were doubled compared to those used in the 2018 and 2019 studies. ^y^ None = water; L12311= *Leuconostoc mesenteroides* strain L12311; and F521 = *Rhizoctonia solani* strain F521 AG2-2 IIIB. ^z^ The two studies were each arranged in a randomized complete block design with eight replications. These studies were analyzed together since the studies were not significantly different (*p* = 0.1887), interactions were not significant (*p* = 0.1471 to 0.8664), and their variances were homogeneous (*p* = 0.6036). *p* > *F* was the probability associated with the *F* value. The means followed by the same letter did not differ significantly based on Fisher’s protected least significant difference (LSD; α = 0.05).

**Table 2:** Differentially expressed (mean normalized FPKM value) sugar beet genes with high expression in the roots at 1-day post-inoculation (dpi) with *Rhizoctonia solani* (Rhi) and/or *Leuconostoc mesenteroides* (Leu). Data are mean of four biological replicates (*p* < 0.05; mock vs. treatment).

Gene_ID	Description	FPKM Mock	FPKM Rhi	FPKM Leu	FPKM Rhi + Leu
EL10Ac3g06968	polygalacturonase inhibitor 1	1.25	435.32	131.58	319.73
EL10Ac6g15542	peroxidase 27	6.39	293.34	5.16	572.29
EL10Ac8g19059	auxin-binding protein ABP19b	7.22	199.84	3.63	214.62
EL10Ac8g19076	auxin-binding protein ABP19b	6.46	129.19	12.12	142.02
EL10Ac8g19060	auxin-binding protein ABP19b	2.84	77.59	2.16	95.43
EL10Ac7g16740	auxin-binding protein ABP19a	0.36	55.57	0.32	16.99
EL10Ac4g08289	auxin-binding protein ABP19a	0.17	10.14	0.13	8.89
EL10Ac4g10349	transmembrane protein 45B	0.98	36.57	3.89	37.68
EL10Ac2g02985	vacuolar amino acid transporter 1	1.89	26.83	1.39	31.65
EL10Ac7g16735	auxin-binding protein ABP19a	0.99	20.75	1.22	13.33
EL10Ac9g22527	auxin-binding protein ABP19b	1.80	17.55	0.43	13.56
EL10Ac3g05264	hypothetical protein	0.93	16.80	1.62	15.15
EL10Ac2g03459	metal tolerance protein 11 isoform X1	18.47	12.63	8.78	0.00
EL10Ac5g13046	putative lipid-binding protein AIR1B	36.36	11.84	265.04	10.81
EL10Ac3g06821	glutamate dehydrogenase B	0.58	10.47	0.07	23.73
EL10Ac9g21246	ubiquitin domain-containing protein DSK2b	0.63	10.46	15.29	0.83
EL10Ac8g20421	auxin-repressed 12.5 kDa protein isoform X1	65.29	5.36	139.86	9.70
EL10Ac8g20056	peroxidase 42	64.02	4.94	72.74	2.84
EL10Ac5g11015	gibberellin-regulated protein 6	31.93	2.71	23.13	2.46
EL10Ac1g00405	aquaporin PIP1-2	43.70	2.39	23.50	2.54
EL10Ac9g22046	aquaporin TIP2-1	27.18	2.08	19.59	1.76
EL10Ac5g11874	probable xyloglucan endotransglucosylase/hydrolase protein 6	21.93	1.59	10.84	1.04
EL10Ac1g00385	40S ribosomal protein S19-2	1.06	1.10	6.83	0.00
EL10Ac5g13042	S-antigen protein	4.31	0.72	34.73	0.87
EL10Ac1g00268	dynamin-related protein 5A	0.26	0.25	11.21	0.43
EL10As13g24048	histone H3.3 isoform X1	0.18	0.33	43.98	0.22

**Table 3:** Differentially expressed (mean normalized FPKM value) *Rhizoctonia solani* (Rhi) genes with high expression in the roots at 1-day post-inoculation (dpi) of sugar beet alone or in combination with *Leuconostoc mesenteroides* (Leu). Data are mean of four biological replicates (*p* < 0.05; mock vs. treatment).

Gene_ID	Description	FPKM Mock	FPKM Rhi	FPKM Rhi + Leu
RSOLAG2-2IIIB_02532	ADP, ATP carrier protein	0	1471.81	1659.11
RSOLAG2-2IIIB_06515	60S ribosomal protein L33-B	0	573.53	696.77
RSOLAG2-2IIIB_06408	elongation factor 1-beta	0	459.05	496.76
RSOLAG2-2IIIB_00056	40S ribosomal protein S2	0	393.52	378.11
RSOLAG2-2IIIB_02188	ribosomal protein S25	0	296.37	432.08
RSOLAG2-2IIIB_02160	40S ribosomal protein S13	0	280.72	327.16
RSOLAG2-2IIIB_03242	glutamine synthetase	0	220.56	274.76
RSOLAG2-2IIIB_00651	uracil permease	0	213.50	125.95
RSOLAG2-2IIIB_02528	40S ribosomal protein S16	0	212.74	322.18
RSOLAG2-2IIIB_04451	D-arabinitol dehydrogenase 1	0	198.54	265.30
RSOLAG2-2IIIB_01551	heat shock 70 kDa protein 2 isoform X3	0	184.56	340.69
RSOLAG2-2IIIB_08269	60S ribosomal protein L17	0	184.52	241.58
RSOLAG2-2IIIB_02789	60S ribosomal protein L11	0	178.30	249.76
RSOLAG2-2IIIB_09687	Mitochondrial phosphate carrier protein	0	160.93	175.34
RSOLAG2-2IIIB_05581	60S ribosomal protein L12	0	142.77	190.19
RSOLAG2-2IIIB_09695	Isocitrate lyase	0	131.99	77.22
RSOLAG2-2IIIB_07137	polygalacturonase At1g48100	0	130.60	97.88
RSOLAG2-2IIIB_01648	hypothetical protein	0	130.45	121.68
RSOLAG2-2IIIB_02803	cytochrome c1, heme protein, mitochondrial	0	124.53	158.66
RSOLAG2-2IIIB_03931	UDP-glucuronic acid decarboxylase 1	0	117.70	65.57
RSOLAG2-2IIIB_01000	ribosome-associated molecular chaperone SSB1	0	116.88	172.23
RSOLAG2-2IIIB_08104	polysaccharide lyase family 14 protein	0	115.50	113.83
RSOLAG2-2IIIB_00954	hypothetical protein CHLRE_17g711200v5	0	114.04	118.90
RSOLAG2-2IIIB_08418	Actin-1	0	113.24	117.12
RSOLAG2-2IIIB_03341	deuterolysin M35 metalloprotease	0	111.56	52.93
RSOLAG2-2IIIB_01353	5-aminolevulinate synthase, mitochondrial	0	103.98	50.89
RSOLAG2-2IIIB_01371	40S ribosomal S3a-2	0	96.74	98.99
RSOLAG2-2IIIB_04036	aspartate aminotransferase, mitochondrial	0	93.66	114.32
RSOLAG2-2IIIB_01383	1,4-alpha-glucan-branching enzyme	0	89.47	109.66
RSOLAG2-2IIIB_06299	glycogen [starch] synthase	0	87.27	71.17
RSOLAG2-2IIIB_06941	Extracellular metalloproteinase	0	85.27	29.03
RSOLAG2-2IIIB_00266	ornithine aminotransferase	0	85.07	80.18
RSOLAG2-2IIIB_06000	proteinase T	0	84.40	48.25
RSOLAG2-2IIIB_05110	polysaccharide lyase family 14 protein	0	84.16	75.84
RSOLAG2-2IIIB_02318	proteasome subunit alpha type 1	0	82.59	55.62
RSOLAG2-2IIIB_02600	D-galacturonate reductase	0	76.26	93.58
RSOLAG2-2IIIB_05867	phosphoenolpyruvate carboxykinase (ATP)	0	75.37	121.92
RSOLAG2-2IIIB_03354	S-adenosylmethionine synthase	0	74.99	125.32
RSOLAG2-2IIIB_10437	hypothetical protein KFL_006780060	0	74.12	93.96
RSOLAG2-2IIIB_05835	transmembrane GTPase fzo1	0	73.38	50.93
RSOLAG2-2IIIB_06333	septin homolog spn4	0	71.73	41.87
RSOLAG2-2IIIB_05426	probable proline-specific permease put4	0	70.32	85.12
RSOLAG2-2IIIB_03646	prohibitin-2	0	68.23	100.93
RSOLAG2-2IIIB_04292	hydroxymethylglutaryl-CoA synthase	0	66.33	64.61
RSOLAG2-2IIIB_02193	60S ribosomal protein L3	0	65.61	65.30
RSOLAG2-2IIIB_00478	ribonucleoside-diphosphate reductase small chain	0	64.76	52.02
RSOLAG2-2IIIB_02703	polyubiquitin isoform X1	0	63.60	99.00
RSOLAG2-2IIIB_09494	Polysaccharide monooxygenase Cel61a	0	62.11	79.78
RSOLAG2-2IIIB_01145	DEAD-box ATP-dependent RNA helicase 15 isoform X1	0	60.80	45.57
RSOLAG2-2IIIB_01675	hypothetical protein	0	57.79	33.54
RSOLAG2-2IIIB_02967	alkaline protease 2	0	55.67	46.90
RSOLAG22IIIB_04341	citrate synthase, mitochondrial	0	54.63	103.13
RSOLAG22IIIB_01161	probable succinate dehydrogenase [ubiquinone] flavoprotein subunit, mitochondrial	0	52.00	60.67
RSOLAG22IIIB_02824	probable glucose transporter rco-3	0	50.26	51.77
RSOLAG22IIIB_01858	copper amine oxidase 1	0	43.72	63.78
RSOLAG22IIIB_01078	superoxide-generating NADPH oxidase heavy chain subunit A	0	41.99	40.58
RSOLAG22IIIB_13422	tubulin alpha chain	0	40.89	32.69
RSOLAG22IIIB_00545	protein sak1	0	29.65	16.20
RSOLAG22IIIB_04811	LOW QUALITY PROTEIN: chitin synthase 8	0	11.79	9.95
RSOLAG22IIIB_03034	hypothetical protein	0	5.75	120.37
RSOLAG22IIIB_02553	60S ribosomal protein L28	0	0.00	112.59
RSOLAG22IIIB_05266	hypothetical protein	0	0.00	69.44
RSOLAG22IIIB_10866	hypothetical protein	0	0.00	182.21

**Table 4:** Differentially expressed (mean normalized FPKM value) *Leuconostoc mesenteroides* (Leu) genes in the roots with high expression at 1-day post-inoculation (dpi) of sugar beet alone or in combination with *Rhizoctonia solani* (Rhi). Data are mean of four biological replicates (*p* < 0.01; mock vs. treatment).

Gene_ID	Description	FPKM Leu	FPKM Rhi + Leu
NH16_RS02710	ATP synthase subunit epsilon	8699.96	15,803.22
NH16_RS04570	translation initiation factor IF-1	3297.98	8390.60
NH16_RS02995	amino acid ABC transporter permease	2220.11	3647.62
NH16_RS08930	30S ribosomal protein S2	1236.86	3206.47
NH16_RS05805	serine hydrolase	1062.12	2102.11
NH16_RS00480	30S ribosome-binding factor RbfA	938.09	2283.04
NH16_RS04555	50S ribosomal protein L15	901.01	2378.87
NH16_RS09120	amino acid ABC transporter permease	830.57	0.00
NH16_RS00875	branched-chain amino acid transport system II carrier protein	812.68	736.23
NH16_RS02465	D-alanyl-lipoteichoic acid biosynthesis protein dltD	196.43	79.37
NH16_RS01415	nicotinate phosphoribosyltransferase	171.19	84.19
NH16_RS00995	YebC/PmpR family DNA-binding transcriptional regulator	161.23	0.00
NH16_RS00265	Histidine–tRNA ligase	75.26	130.30
NH16_RS05780	Phenylalanine–tRNA ligase subunit beta	74.81	122.54
NH16_RS05900	transcription elongation factor GreA	70.30	71.75
NH16_RS04250	nucleoid-associated protein	62.71	0.00
NH16_RS04785	DUF2969 domain-containing protein	50.91	68.67
NH16_RS03560	putative sulfate exporter family transporter	50.52	52.31
NH16_RS04255	DUF1810 family protein	42.28	8.53
NH16_RS04620	energy-coupling factor transporter transmembrane protein EcfT	41.54	21.49
NH16_RS01695	glutamyl aminopeptidase	37.59	36.52
NH16_RS00200	rRNA pseudouridine synthase	30.08	31.72
NH16_RS07185	AAA family ATPase	29.56	12.64
NH16_RS08255	[citrate (pro-3S)-lyase] ligase	15.74	68.51
NH16_RS02495	alpha/beta hydrolase	12.70	54.78
NH16_RS04445	ABC transporter permease	10.18	217.69

## Data Availability

The authors declare availability of data and material upon request.

## Supplementary Materials

The following supporting information can be downloaded at: https://www.mdpi.com/article/10.3390/ijms23031366/s1.
