# Supplementary material for: Cell-Wall-Degrading Enzymes-Related Genes Originating from Rhizoctonia solani Increase Sugar Beet Root Damage in the Presence of Leuconostoc mesenteroides
Source: Int J Mol Sci. 2022 Jan 25;23(3):1366. doi: 10.3390/ijms23031366 (PMC8835807; doi:10.3390/ijms23031366)
Supplement: Supplementary file 1 [file ijms-23-01366-s001.zip › ijms-1547189-supplementary.pdf]

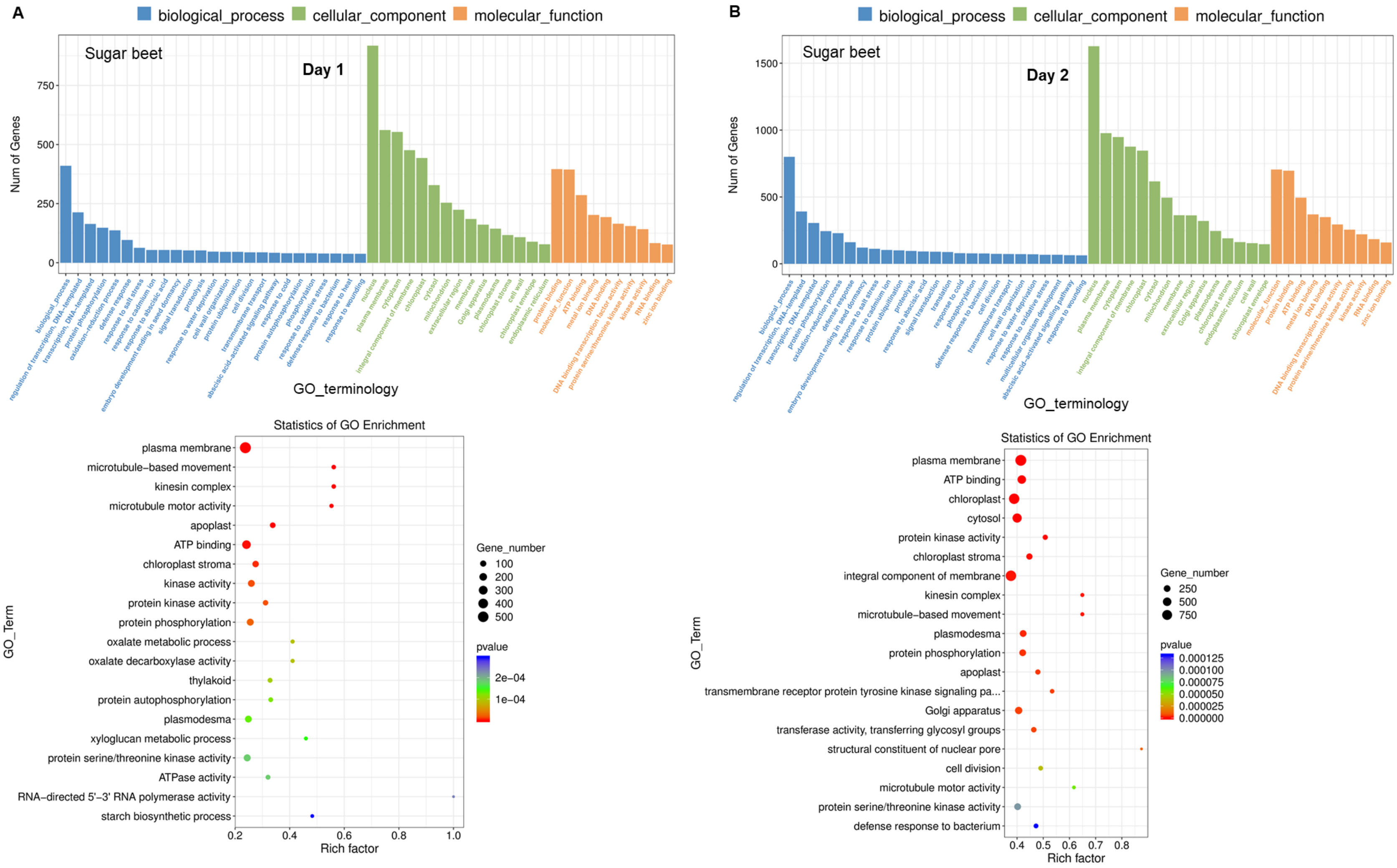

Figure S1. (continued)

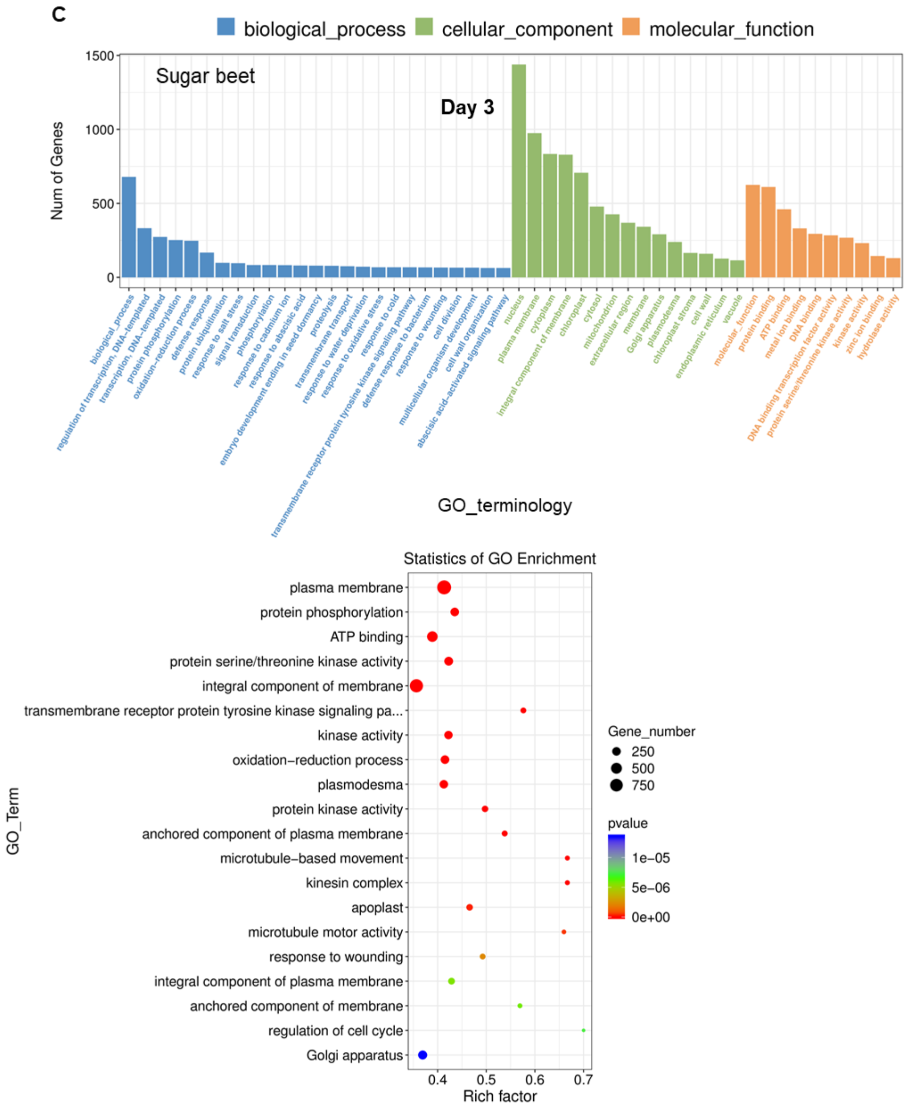

Figure S1. Gene ontology (GO) of differentially expressed sugar beet genes show a distinct pattern with different infection stages during interaction with *Rhizoctonia solani* and *Leuconostoc mesenteroides*. (A) 1 dpi, (B) 2 dpi, and (C) 3 dpi.

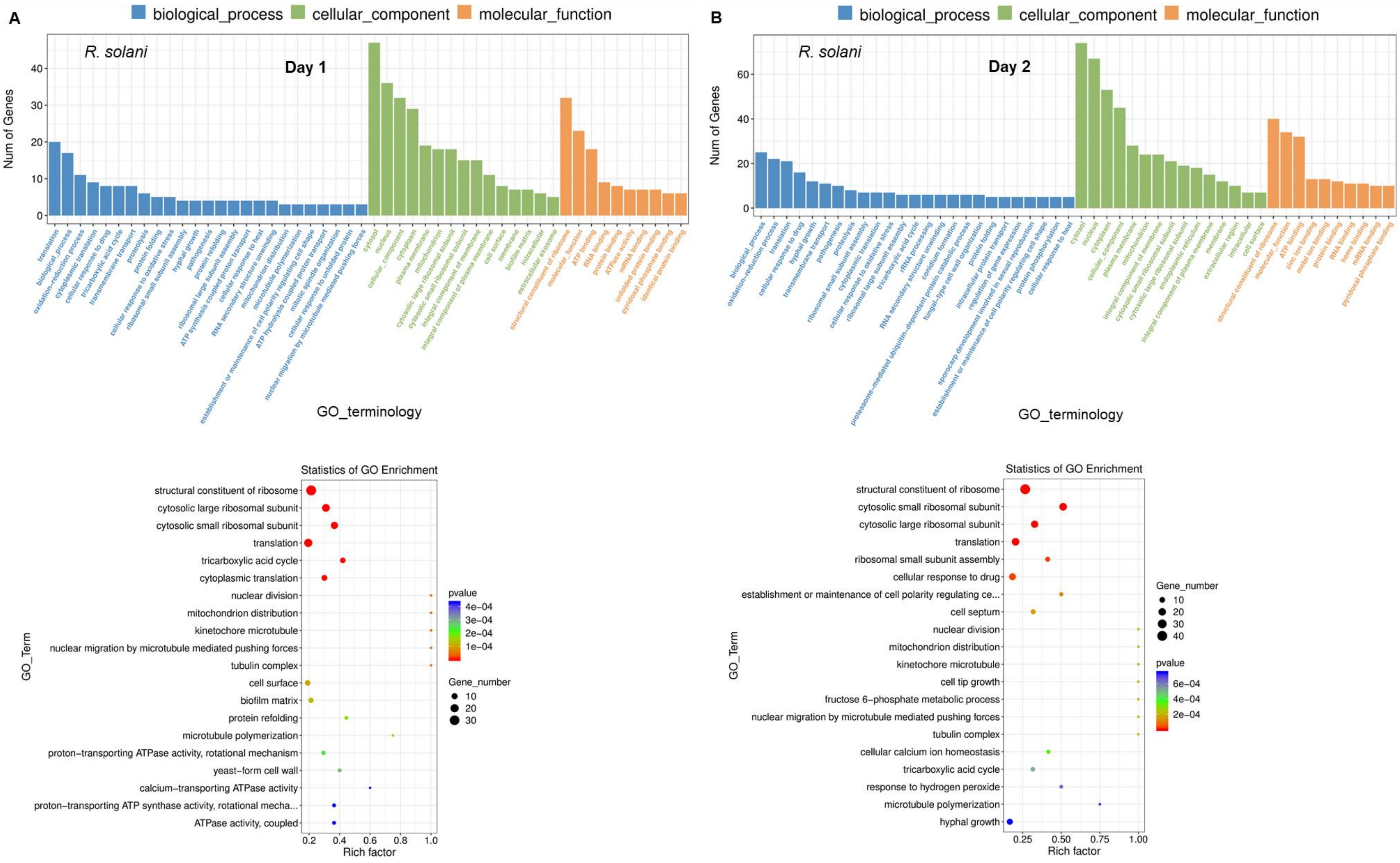

Figure S2. (continued)

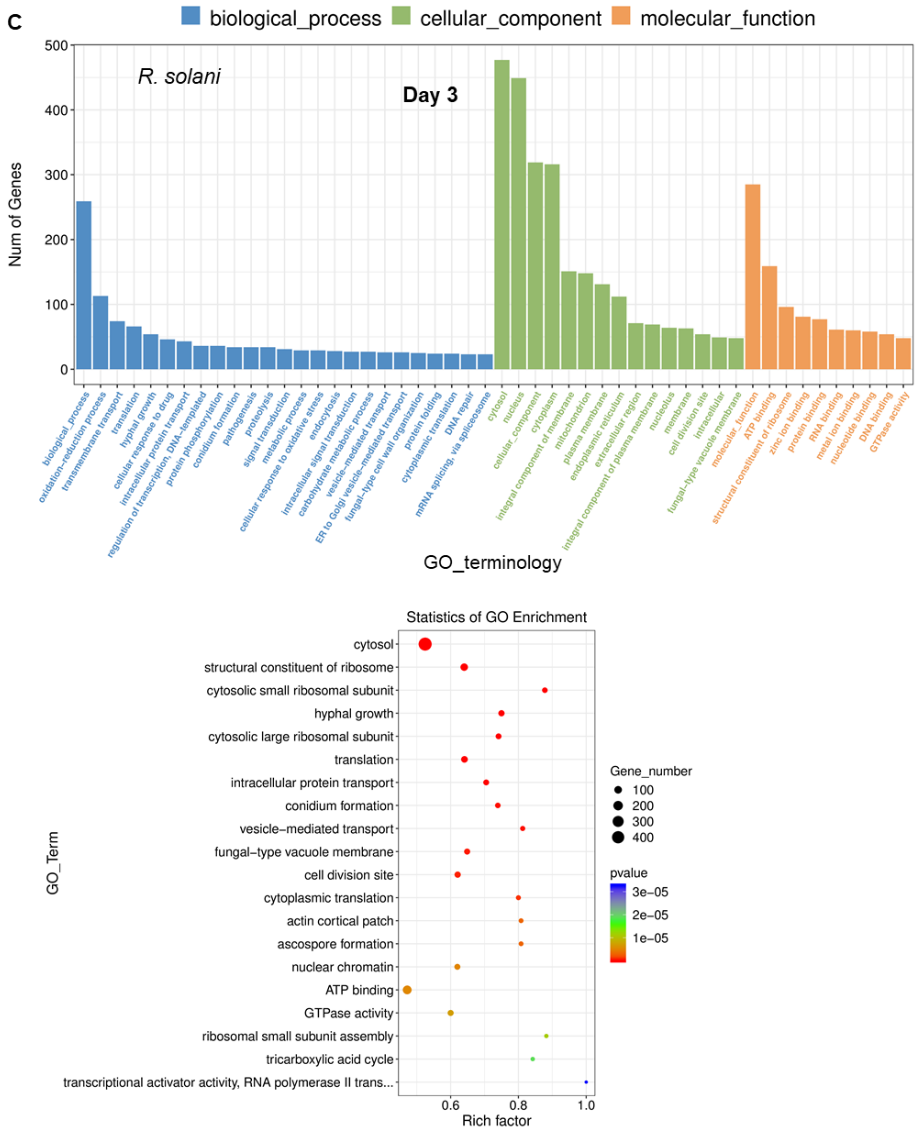

Figure S2. Gene ontology (GO) of differentially expressed *Rhizoctonia solani* genes show a distinct pattern with different infection stages during interaction with sugar beet and *Leuconostoc mesenteroides*. (A) 1 dpi, (B) 2 dpi, and (C) 3 dpi.

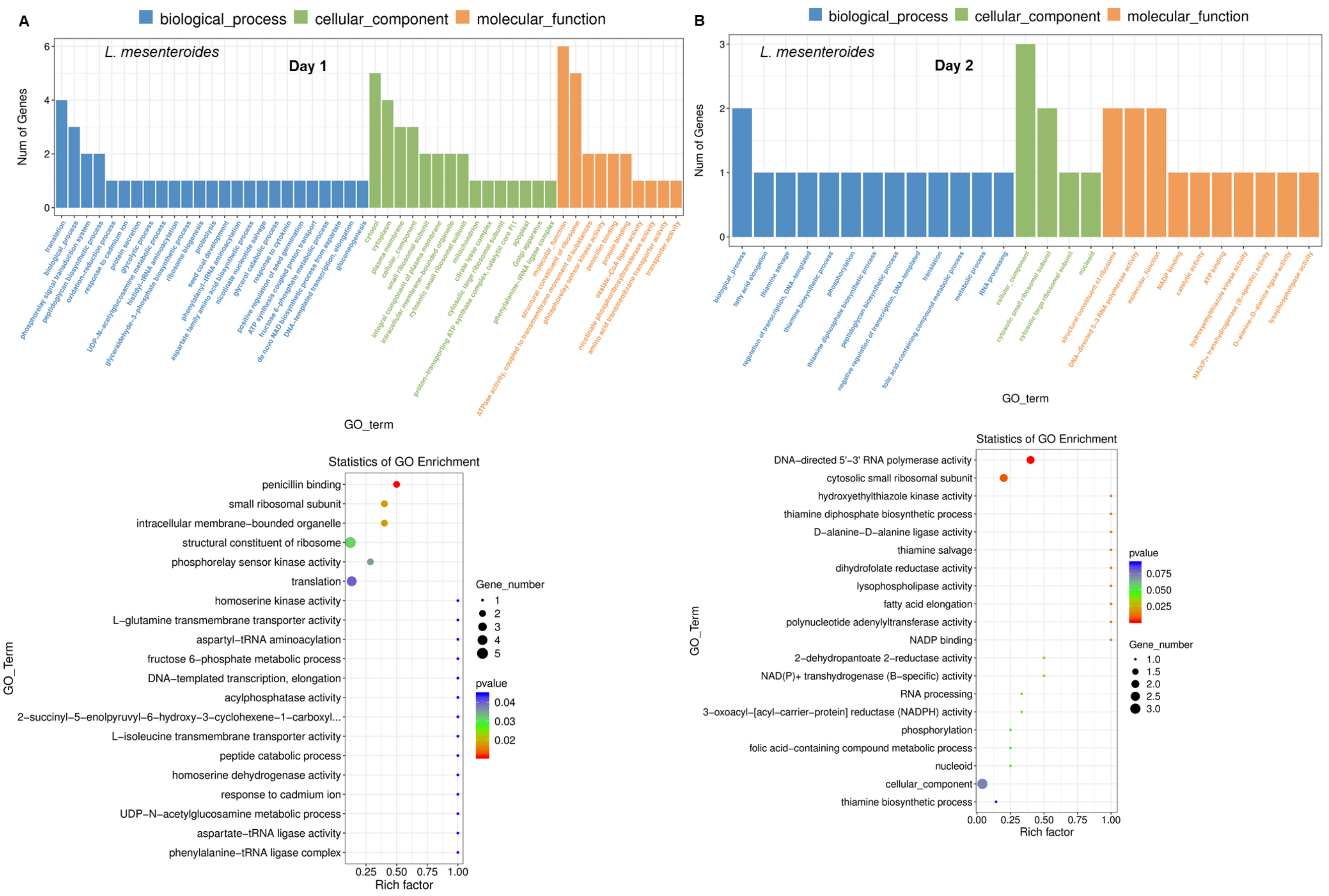

Figure S3. (continued)

C

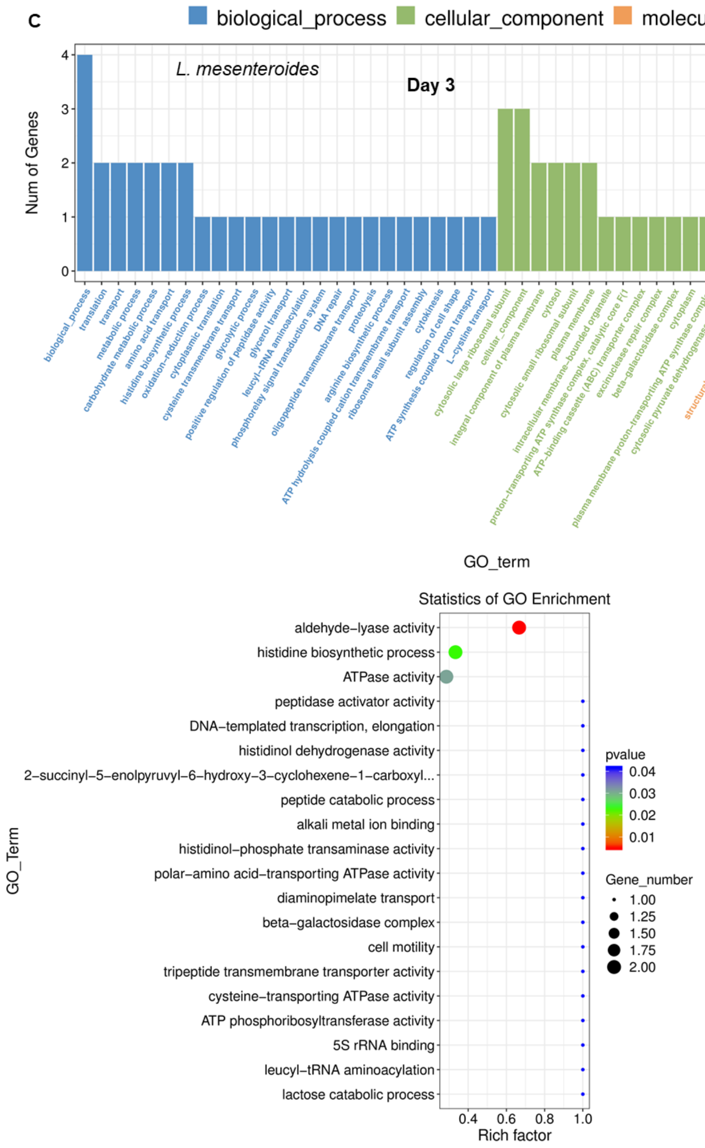

Figure S3. Gene ontology (GO) of differentially expressed *Leuconostoc mesenteroides* genes show a distinct pattern with different infection stages during interaction with sugar beet and *Rhizoctonia solani*. (A) 1 dpi, (B) 2 dpi, and (C) 3 dpi.

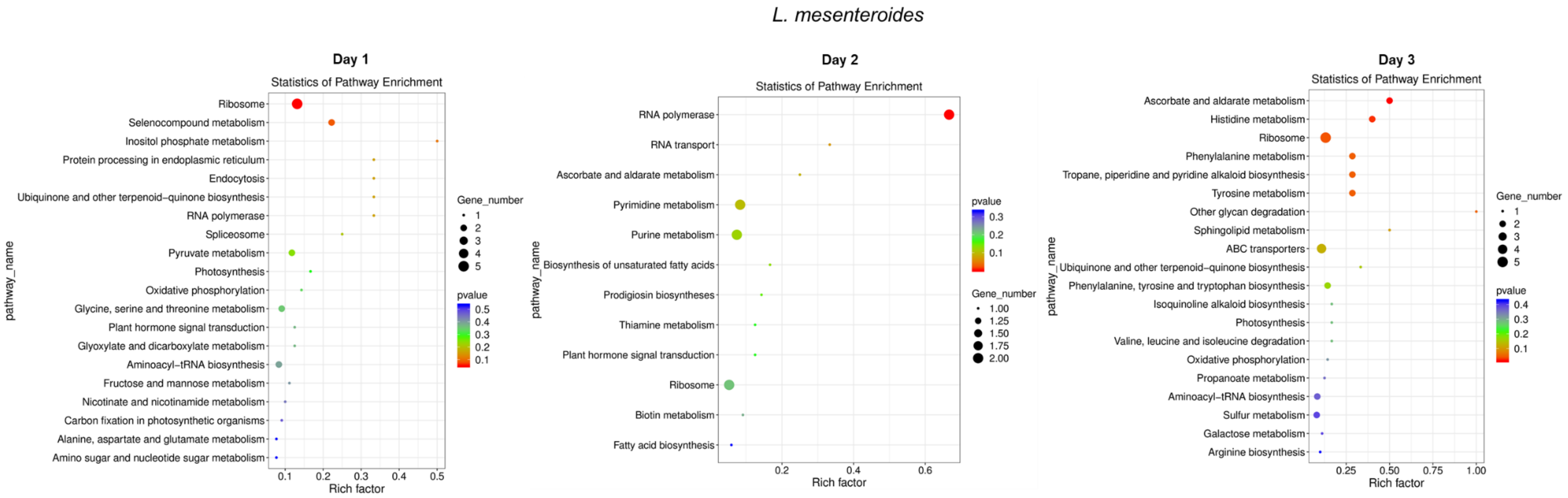

Figure S4. Pathway enrichment of *Leuconostoc mesenteroides* genes show differential responses with infection stages during interaction with sugar beet and *Rhizoctonia solani*. Kyoto Encyclopedia of Genes and Genomes (KEGG) enrichment of *R. solani* genes at (A) 1 dpi, (B) 2 dpi, and (C) 3 dpi. Data are mean of 4 biological replicates.

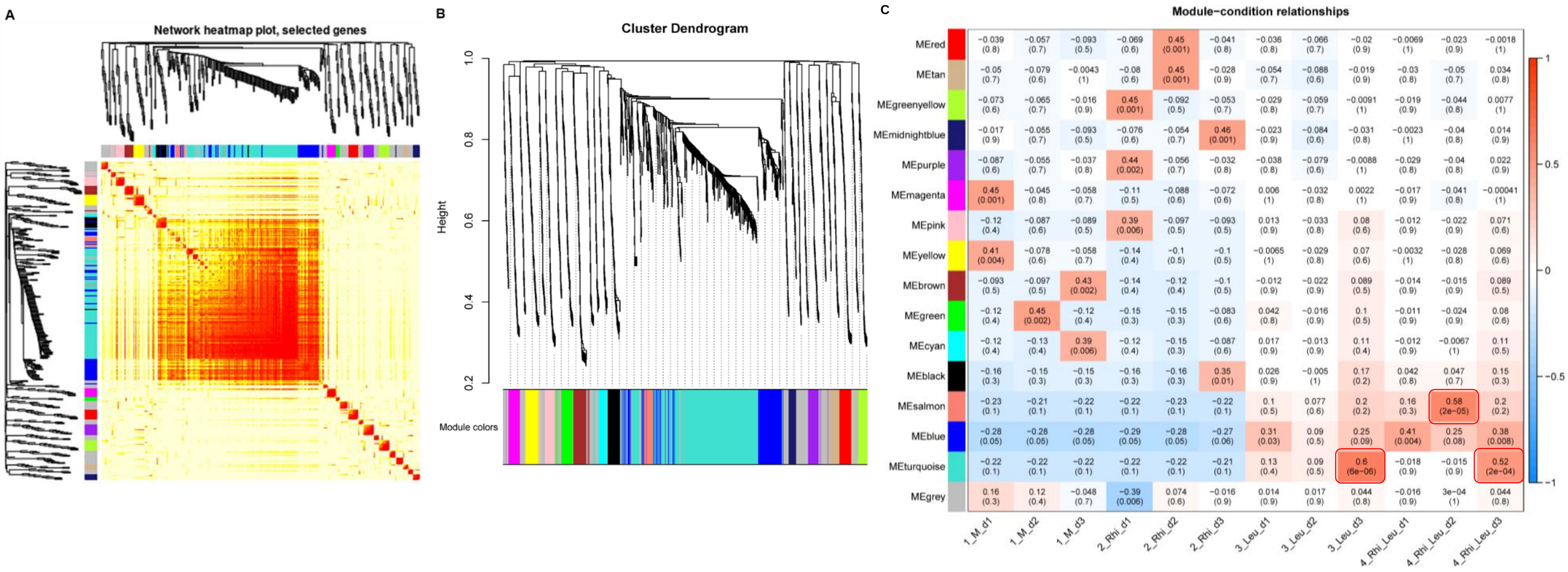

Figure S5. Weighted gene co-expression network analysis (WGCNA) of *Leuconostoc mesenteroides* genes during interaction with sugar beet in presence or absence of *R. solani* show distinct clustering pattern with infection stages and treatment type. (A) Heatmap showing network of differentially expressed selected genes, (B) gene cluster dendrogram, and (C) module-condition relationship. Data are Mean of 4 biological replicates. Modules with high correlation values (>0.5) and significance (p < 0.05) are boxed with red rectangles.

Table S1. Differentially expressed (mean normalized FPKM value) representative sugar beet genes with high expression in the roots at 2-day post inoculation (dpi) with *Rhizoctonia solani* (Rhi) and *Leuconostoc mesenteroides* (Leu). Data are Mean of 4 biological replicates ($p < 0.05$; mock vs. treatment).

Gene_ID	Description	FPKM Mock	FPKM Rhi	FPKM Leu	FPKM Rhi+Leu
EL10Ac8g19059	auxin-binding protein ABP19b	1.75	313.32	4.24	299.88
EL10Ac6g15542	peroxidase 27	0.93	306.29	2.28	503.30
EL10Ac6g14807	E3 ubiquitin-protein ligase ATL31	12.09	306.12	35.44	253.93
EL10Ac4g09723	4,5-DOPA dioxygenase extradiol	4.99	170.08	27.78	175.31
EL10Ac8g19060	auxin-binding protein ABP19b	0.92	164.02	2.64	170.26
EL10Ac4g09811	uncharacterized protein	6.49	156.12	21.31	145.69
EL10Ac8g19057	auxin-binding protein ABP19b	1.62	151.66	1.44	167.91
EL10Ac8g19078	auxin-binding protein ABP19b	1.10	128.44	2.59	104.51
EL10Ac8g19076	auxin-binding protein ABP19b	1.70	95.00	4.34	96.13
EL10Ac7g17522	hypothetical protein	3.91	87.92	10.30	76.25
EL10Ac8g20260	Pollen proteins Ole e I	3.18	80.71	17.03	95.11
EL10Ac4g10349	transmembrane protein 45B	0.87	79.63	5.61	69.48
EL10Ac2g03442	zinc finger protein ZAT12	2.60	79.32	14.90	69.93
EL10Ac4g09030	probable glutathione S-transferase parC	0.00	61.54	0.30	56.53
EL10Ac5g11004	glutathione S-transferase U9	3.20	52.35	8.82	52.55
EL10Ac8g18508	unknown function	2.11	50.11	6.81	54.25
EL10Ac9g22251	probable indole-3-acetic acid-amido synthetase GH3.1	0.89	49.43	5.32	48.43
EL10Ac9g21784	allene oxide synthase	4.79	47.97	5.82	58.12
EL10Ac6g14665	hypothetical protein	0.30	43.59	0.73	35.95
EL10Ac9g22526	auxin-binding protein ABP19a	0.40	36.23	2.35	29.55
EL10Ac2g02985	vacuolar amino acid transporter 1	0.98	28.12	0.72	31.28
EL10Ac9g22527	auxin-binding protein ABP19b	0.86	25.08	0.57	27.63
EL10Ac5g10581	UDP-glycosyltransferase 74F2	0.56	20.58	3.38	23.78
EL10Ac2g04904	laccase-12	0.00	18.50	14.61	14.64
EL10Ac9g22620	benzyl alcohol O-benzoyltransferase	0.31	15.14	2.47	24.50
EL10Ac4g08289	auxin-binding protein ABP19a	0.68	14.97	1.49	17.42
EL10Ac7g16738	auxin-binding protein ABP19a	0.10	14.86	0.46	18.41
EL10Ac5g11314	mitochondrial phosphate carrier protein 3, mitochondrial	0.00	14.65	0.98	3.65
EL10Ac3g05264	unknown function	0.05	13.99	0.48	15.11
EL10Ac1g00741	fasciclin-like arabinogalactan protein 21	0.23	10.44	0.54	8.32
EL10Ac8g19379	probable nucleolar protein 5-2	0.00	8.00	2.73	8.16
EL10Ac5g11015	gibberellin-regulated protein 6	54.85	6.24	39.32	1.81
EL10Ac2g04142	ethylene-responsive transcription factor ERF003	30.66	4.41	25.69	0.00
EL10Ac7g16859	protein SRG1	0.00	3.40	2.16	28.20
EL10Ac6g15595	probable leucine-rich repeat receptor-like protein kinase At1g35710	38.48	3.06	26.07	0.95
EL10Ac5g11344	mitochondrial import inner membrane translocase subunit TIM8	3.64	0.00	20.83	0.00
EL10Ac8g20056	peroxidase 42	59.37	2.42	52.60	0.79
EL10Ac9g22046	aquaporin TIP2-1	54.03	1.34	22.50	0.30
EL10Ac4g10104	endoglucanase 8	8.75	0.72	4.82	0.11
EL10Ac3g05664	V-type proton ATPase subunit C	0.00	0.00	39.21	22.64

Table S2. Differentially expressed (mean normalized FPKM value) representative sugar beet genes with high expression in the roots at 3-day post inoculation (dpi) with *Rhizoctonia solani* (Rhi) and *Leuconostoc mesenteroides* (Leu). Data are Mean of 4 biological replicates ($p < 0.05$; mock vs. treatment).

Gene_ID	Description	FPKM Mock	FPKM Rhi	FPKM Leu	FPKM Rhi+Leu
EL10Ac7g15862	peroxidase 4	14.12	885.32	253.33	813.73
EL10Ac4g08263	pathogenesis-related protein PR-4	14.72	718.47	550.88	1244.01
EL10Ac9g20649	tyrosine/DOPA decarboxylase 1	8.01	616.73	96.31	511.71
EL10Ac5g12050	uncharacterized acetyltransferase At3g50280	9.84	514.73	74.23	438.68
EL10Ac9g21173	basic 7S globulin	4.97	301.84	88.11	198.21
EL10Ac4g09723	4,5-DOPA dioxygenase extradiol	2.89	230.43	47.74	295.29
EL10As3g23352	2-hydroxyisoflavanone dehydratase	6.20	220.86	59.05	265.30
EL10Ac7g17571	ethylene-responsive transcription factor ERF073	2.10	212.62	45.94	241.78
EL10Ac6g15542	peroxidase 27	0.51	206.15	14.44	105.50
EL10Ac8g19057	auxin-binding protein ABP19b	0.19	165.58	10.12	131.31
EL10Ac3g07123	zeatin O-glucosyltransferase	4.23	132.63	28.26	157.86
EL10Ac9g21828	unknown function	5.15	129.09	18.50	115.18
EL10Ac8g18457	hypothetical protein BVRB_1g000360	0.14	127.41	21.53	65.84
EL10Ac6g14807	E3 ubiquitin-protein ligase ATL31	2.42	118.77	31.29	171.74
EL10Ac8g19059	auxin-binding protein ABP19b	0.12	118.39	11.24	118.62
EL10Ac4g09811	unknown function	1.98	100.72	16.59	72.94
EL10Ac2g03442	zinc finger protein ZAT12	2.17	86.31	31.23	123.62
EL10Ac4g09030	probable glutathione S-transferase parC	0.00	81.46	26.19	71.48
EL10Ac1g01145	hypothetical protein	4.14	80.69	12.06	80.89
EL10Ac9g22781	EF hand domain containing protein	1.46	80.54	44.69	85.91
EL10Ac4g09467	berberine bridge enzyme-like 8	1.96	76.03	19.63	81.69
EL10Ac5g12016	probable aminotransferase TAT2	0.07	74.03	15.75	52.57
EL10Ac8g18390	hypothetical protein BVRB_8g197390	1.86	73.65	11.58	70.25
EL10Ac4g08019	FAD-dependent urate hydroxylase	0.33	72.88	12.50	59.42
EL10Ac5g11908	ubiquinol oxidase 2, mitochondrial-like	0.65	72.17	15.53	102.16
EL10Ac6g15677	probable indole-3-acetic acid-amido synthetase GH3.1	0.28	67.91	11.34	59.32
EL10Ac8g19060	auxin-binding protein ABP19b	0.04	65.17	6.07	73.88
EL10Ac4g10349	transmembrane protein 45B	0.98	62.64	13.40	94.15
EL10Ac8g19076	auxin-binding protein ABP19b	0.25	57.51	5.74	32.33
EL10Ac9g21784	allene oxide synthase	3.63	53.89	10.42	86.45
EL10Ac1g00584	putative germin-like protein 2-1	1.10	50.63	21.21	91.12
EL10Ac6g14837	anthocyanidin 3-O-glucosyltransferase 2	1.99	50.54	10.97	46.00
EL10Ac6g13535	probable WRKY transcription factor 13	0.00	48.75	12.30	0.00
EL10Ac1g01083	hypothetical protein	0.05	48.54	3.63	16.42
EL10Ac9g22620	benzyl alcohol O-benzoyltransferase	0.42	48.34	5.15	39.14
EL10Ac1g02328	cytochrome P450 81E8	0.14	45.89	9.24	48.31
EL10Ac5g11004	glutathione S-transferase U9	1.79	43.96	13.02	69.96
EL10Ac7g15909	UDP-glycosyltransferase 87A2	2.13	43.56	11.83	41.63
EL10Ac7g17719	alcohol dehydrogenase class-3	22.01	42.17	0.00	28.46
EL10Ac9g22251	probable indole-3-acetic acid-amido synthetase GH3.1	0.22	37.41	6.81	32.47
EL10Ac5g10581	UDP-glycosyltransferase 74F2	0.52	37.25	6.21	53.16
EL10Ac5g12393	B3 domain-containing protein	0.15	34.41	5.96	24.30
EL10Ac7g16740	Auxin-binding protein ABP19a	0.04	32.46	1.51	35.59
EL10Ac7g16199	Mitogen-activated protein kinase A	0.43	32.40	5.19	27.32
EL10Ac1g01588	ethylene-responsive transcription factor ABR1	0.24	31.84	5.50	21.70
EL10Ac2g03379	gibberellin-regulated protein 14	137.48	6.24	31.34	3.57
EL10Ac8g20056	peroxidase 42	273.12	5.18	55.45	2.50

EL10Ac7g16456	dormancy-associated protein homolog 4	72.46	4.49	38.97	4.04
EL10Ac1g00405	aquaporin PIP1-2	76.51	3.44	21.43	1.26
EL10Ac2g04670	probable lipid transfer	12.03	3.32	3.24	0.01
EL10Ac4g08792	hypothetical protein	46.72	3.08	4.33	1.24
EL10As5g23597	galactinol synthase BvRafS1	76.57	2.99	15.54	6.93
EL10Ac9g22046	aquaporin TIP2-1	103.65	1.33	16.63	1.08

Table S3. Differentially expressed (mean normalized FPKM value) representative *Rhizoctonia solani* (Rhi) genes with high expression in the roots at 2-day post inoculation (dpi) of sugar beet alone or in combination with *Leuconostoc mesenteroides* (Leu). Data are Mean of 4 biological replicates ($p < 0.05$; mock vs. treatment).

Gene_ID	Description	FPKM Mock	FPKM Rhi	FPKM Rhi + Leu
RSOLAG2-2IIIB_03040	PREDICTED: 40S ribosomal protein S28-B	0	857.65	650.71
RSOLAG2-2IIIB_06408	elongation factor 1-beta	0	558.21	516.16
RSOLAG2-2IIIB_05721	hypothetical protein	0	542.57	420.48
RSOLAG2-2IIIB_04451	D-arabinitol dehydrogenase 1	0	476.89	362.08
RSOLAG2-2IIIB_02188	ribosomal protein S25	0	417.35	444.79
RSOLAG2-2IIIB_01577	peptidyl-Lys metalloendopeptidase	0	388.03	268.59
RSOLAG2-2IIIB_00954	hypothetical protein	0	355.74	257.57
RSOLAG2-2IIIB_02160	40S ribosomal protein S13	0	316.53	287.27
RSOLAG2-2IIIB_03341	deuterolysin M35 metalloprotease	0	314.37	251.86
RSOLAG2-2IIIB_02528	40S ribosomal protein S16	0	302.32	285.81
RSOLAG2-2IIIB_00651	uracil permease	0	290.13	196.73
RSOLAG2-2IIIB_10173	proteinase T-like	0	289.13	258.68
RSOLAG2-2IIIB_01551	heat shock 70 kDa protein 2 isoform X3	0	272.63	294.80
RSOLAG2-2IIIB_04169	guanine nucleotide-binding protein subunit beta-like protein	0	256.42	220.32
RSOLAG2-2IIIB_02789	60S ribosomal protein L11	0	246.80	255.13
RSOLAG2-2IIIB_01383	1,4-alpha-glucan-branching enzyme	0	231.44	185.75
RSOLAG2-2IIIB_01895	40S ribosomal protein S10-A	0	199.05	185.24
RSOLAG2-2IIIB_02293	alkali-sensitive linkage protein 1	0	187.56	154.03
RSOLAG2-2IIIB_02803	cytochrome c1, heme protein, mitochondrial	0	185.37	179.62
RSOLAG2-2IIIB_06812	probable metal-nicotianamine transporter YSL4	0	174.82	114.59
RSOLAG2-2IIIB_03931	PREDICTED: UDP-glucuronic acid decarboxylase 1	0	168.45	121.92
RSOLAG2-2IIIB_05581	60S ribosomal protein L12	0	166.98	182.31
RSOLAG2-2IIIB_10727	proteinase T	0	166.16	122.95
RSOLAG2-2IIIB_01858	copper amine oxidase 1	0	155.42	96.65
RSOLAG2-2IIIB_02703	polyubiquitin isoform X1	0	149.73	116.27
RSOLAG2-2IIIB_02600	D-galacturonate reductase	0	149.65	133.18
RSOLAG2-2IIIB_08104	polysaccharide lyase family 14 protein	0	146.87	127.86
RSOLAG2-2IIIB_07842	probable peptide transporter ptr2	0	125.36	86.94
RSOLAG2-2IIIB_00696	5-methyltetrahydropteroyltriglutamate--homocysteine methyltransferase	0	117.05	100.01
RSOLAG2-2IIIB_10920	ATP synthase subunit 5, mitochondrial	0	116.89	124.88
RSOLAG2-2IIIB_01371	40S ribosomal S3a-2	0	115.05	99.06
RSOLAG2-2IIIB_10729	proteinase T	0	113.55	92.14
RSOLAG2-2IIIB_10437	hypothetical protein	0	113.36	109.83
RSOLAG2-2IIIB_05110	polysaccharide lyase family 14 protein	0	110.07	75.85
RSOLAG2-2IIIB_03354	S-adenosylmethionine synthase	0	109.78	92.60
RSOLAG2-2IIIB_11563	alanine--glyoxylate aminotransferase 1	0	106.83	76.28
RSOLAG2-2IIIB_06299	glycogen [starch] synthase	0	106.76	88.68
RSOLAG2-2IIIB_00266	ornithine aminotransferase	0	106.22	84.60
RSOLAG2-2IIIB_03646	prohibitin-2	0	104.01	112.17
RSOLAG2-2IIIB_05932	putative sterigmatocystin biosynthesis protein stcT	0	101.85	84.79

RSOLAG2-2IIIB_04036	aspartate aminotransferase, mitochondrial	0	100.01	100.13
RSOLAG2-2IIIB_04341	citrate synthase, mitochondrial	0	99.93	101.63
RSOLAG2-2IIIB_01965	60S acidic ribosomal protein P0	0	98.92	0.00
RSOLAG2-2IIIB_02063	thiamine pathway transporter THI73	0	97.32	71.41
RSOLAG2-2IIIB_09088	nitrite reductase [NAD(P)H]	0	95.66	67.71
RSOLAG2-2IIIB_06333	septin homolog spn4	0	89.00	68.97
RSOLAG2-2IIIB_00596	protein disulfide-isomerase	0	83.16	68.02
RSOLAG2-2IIIB_03614	mitochondrial outer membrane protein porin	0	78.91	75.46
RSOLAG2-2IIIB_02193	60S ribosomal protein L3	0	74.10	56.56
RSOLAG2-2IIIB_01145	DEAD-box ATP-dependent RNA helicase 15 isoform X1	0	71.06	63.31
RSOLAG2-2IIIB_00730	meiotic sister chromatid recombination protein 1	0	69.47	48.73
RSOLAG2-2IIIB_08266	mitochondrial processing peptidase	0	69.27	80.86
RSOLAG2-2IIIB_05835	transmembrane GTPase fzo1	0	69.14	66.16
RSOLAG2-2IIIB_04292	hydroxymethylglutaryl-CoA synthase	0	67.75	58.57
RSOLAG2-2IIIB_09695	hypothetical protein TanjilG_03100	0	66.09	71.33
RSOLAG2-2IIIB_01496	L-galactonate dehydratase	0	65.68	55.07
RSOLAG2-2IIIB_05058	carboxypeptidase Y homolog A	0	65.00	49.72
RSOLAG2-2IIIB_00470	fructose-1,6-bisphosphatase, cytosolic	0	62.20	74.85
RSOLAG2-2IIIB_04062	phosphoglucomutase	0	60.31	53.13
RSOLAG2-2IIIB_00164	hypothetical protein	0	58.93	62.18
RSOLAG2-2IIIB_10184	protein-glutamate O-methyltransferase C1393.13	0	52.03	52.25
RSOLAG2-2IIIB_01353	5-aminolevulinate synthase, mitochondrial	0	51.86	62.62
RSOLAG2-2IIIB_09494	polysaccharide monooxygenase Cel61a	0	48.71	76.74
RSOLAG2-2IIIB_05616	putative protein C32A11,02c	0	42.27	50.56

Table S4. Differentially expressed (mean normalized FPKM value) representative *Rhizoctonia solani* (Rhi) genes with high expression in the roots at 3-day post inoculation (dpi) of sugar beet alone or in combination with *Leuconostoc mesenteroides* (Leu). Data are Mean of 4 biological replicates ($p < 0.05$; mock vs. treatment).

Gene_ID	Description	FPKM Mock	FPKM Rhi	FPKM Rhi + Leu
RSOLAG2-2IIIB_03040	40S ribosomal protein S28-B	0	790.05	984.05
RSOLAG2-2IIIB_08570	40S ribosomal protein S12	0	585.18	548.73
RSOLAG2-2IIIB_03281	60S ribosomal protein	0	482.21	367.00
RSOLAG2-2IIIB_01895	40S ribosomal protein S10-A	0	407.43	363.67
RSOLAG2-2IIIB_07009	60S ribosomal protein L37a	0	406.65	498.76
RSOLAG2-2IIIB_02160	40S ribosomal protein S13	0	387.96	399.76
RSOLAG2-2IIIB_02803	cytochrome c1, heme protein, mitochondrial	0	372.68	340.36
RSOLAG2-2IIIB_04169	guanine nucleotide-binding protein subunit beta-like protein	0	361.58	384.15
RSOLAG2-2IIIB_00227	40S ribosomal protein S23	0	360.07	338.87
RSOLAG2-2IIIB_01648	hypothetical protein	0	340.07	225.35
RSOLAG2-2IIIB_01383	1,4-alpha-glucan-branching enzyme	0	323.52	269.80
RSOLAG2-2IIIB_12410	predicted protein	0	289.48	245.80
RSOLAG2-2IIIB_01426	40S ribosomal S11	0	254.86	247.07
RSOLAG2-2IIIB_01011	60S ribosomal protein L26-1	0	244.09	230.07
RSOLAG2-2IIIB_02789	60S ribosomal protein L11	0	239.91	268.88
RSOLAG2-2IIIB_02600	D-galacturonate reductase	0	239.67	179.43
RSOLAG2-2IIIB_05426	probable proline-specific permease put4	0	232.88	210.87
RSOLAG2-2IIIB_00171	40S ribosomal protein S0	0	232.66	216.65
RSOLAG2-2IIIB_04827	40S ribosomal protein S17-A	0	208.37	190.71
RSOLAG2-2IIIB_11563	alanine--glyoxylate aminotransferase 1	0	182.37	98.63
RSOLAG2-2IIIB_10920	ATP synthase subunit 5, mitochondrial	0	180.73	180.41
RSOLAG2-2IIIB_04341	citrate synthase, mitochondrial	0	170.31	207.04
RSOLAG2-2IIIB_04521	succinate dehydrogenase [ubiquinone] iron-sulfur subunit, mitochondrial	0	157.77	132.74
RSOLAG2-2IIIB_04442	adenosylhomocysteinase	0	156.80	111.10
RSOLAG2-2IIIB_03354	S-adenosylmethionine synthase	0	156.08	168.54
RSOLAG2-2IIIB_00091	cytochrome c peroxidase, mitochondrial	0	147.49	119.77
RSOLAG2-2IIIB_00338	2-methylcitrate dehydratase	0	146.97	126.78
RSOLAG2-2IIIB_01122	hypothetical protein	0	143.68	81.08
RSOLAG2-2IIIB_03646	prohibitin-2	0	141.73	148.21
RSOLAG2-2IIIB_02193	60S ribosomal protein L3	0	140.21	130.11
RSOLAG2-2IIIB_03237	isocitrate dehydrogenase [NAD] subunit 1, mitochondrial	0	135.64	124.05
RSOLAG2-2IIIB_00478	ribonucleoside-diphosphate reductase small chain	0	134.49	112.01
RSOLAG2-2IIIB_00347	hypothetical protein	0	125.42	93.25
RSOLAG2-2IIIB_01586	putative 4-hydroxy-2-oxoglutarate aldolase, mitochondrial	0	122.80	67.51
RSOLAG2-2IIIB_01940	PREDICTED: ERBB-3 BINDING PROTEIN 1 isoform X2	0	111.97	108.81
RSOLAG2-2IIIB_03917	isocitrate dehydrogenase [NAD] subunit 2, mitochondrial	0	110.83	100.16
RSOLAG2-2IIIB_01921	hypothetical protein	0	107.88	94.54
RSOLAG2-2IIIB_05058	carboxypeptidase Y homolog A	0	107.10	61.79
RSOLAG2-2IIIB_05998	mitochondrial arginine transporter BAC2	0	106.57	102.01
RSOLAG2-2IIIB_00470	fructose-1,6-bisphosphatase, cytosolic	0	105.76	85.41
RSOLAG2-2IIIB_00587	hypothetical protein	0	101.15	72.33

RSOLAG2-2IIIB_01836	phosphatidate cytidylyltransferase, mitochondrial	0	99.81	87.73
RSOLAG2-2IIIB_01145	DEAD-box ATP-dependent RNA helicase 15 isoform X1	0	98.98	74.84
RSOLAG2-2IIIB_07551	adenosine kinase	0	96.93	92.89
RSOLAG2-2IIIB_00740	hypothetical protein	0	94.39	87.42
RSOLAG2-2IIIB_08901	ornithine decarboxylase	0	92.42	86.07
RSOLAG2-2IIIB_10437	hypothetical protein	0	91.44	88.90
RSOLAG2-2IIIB_06941	extracellular metalloproteinase	0	91.31	44.95
RSOLAG2-2IIIB_06059	PREDICTED: putative UDP-sugar transporter DDB_G0278631	0	89.17	82.63
RSOLAG2-2IIIB_02703	polyubiquitin isoform X1	0	87.54	91.93
RSOLAG2-2IIIB_00372	heat shock protein hsp88	0	87.03	71.40
RSOLAG2-2IIIB_01675	hypothetical protein	0	83.11	63.34
RSOLAG2-2IIIB_06924	serine palmitoyltransferase 2	0	81.61	67.21
RSOLAG2-2IIIB_01539	polyadenylate-binding protein, cytoplasmic and nuclear	0	80.34	37.47
RSOLAG2-2IIIB_08957	1,3-beta-glucan synthase component FKS1	0	75.46	55.92
RSOLAG2-2IIIB_01504	glutamine--fructose-6-phosphate aminotransferase [isomerizing]	0	74.36	58.72
RSOLAG2-2IIIB_01939	homocitrate synthase, mitochondrial	0	70.90	103.33
RSOLAG2-2IIIB_05421	RNA-binding post-transcriptional regulator cip2	0	70.44	59.18
RSOLAG2-2IIIB_00123	putative mitochondrial chaperone BCS1-B	0	70.21	53.06
RSOLAG2-2IIIB_03937	nucleoside diphosphate kinase	0	63.78	52.53
RSOLAG2-2IIIB_03408	exportin-1	0	63.75	44.87
RSOLAG2-2IIIB_07909	V-type proton ATPase subunit B	0	63.50	53.90
RSOLAG2-2IIIB_01118	pyruvate decarboxylase	0	59.35	99.58

Table S5. Differentially expressed (mean normalized FPKM value) representative *Leuconostoc mesenteroides* (Leu) genes with high expression in the roots at 2-day post inoculation (dpi) of sugar beet alone or in combination with *Rhizoctonia solani* (Rhi). Data are Mean of 4 biological replicates ($p < 0.01$; mock vs. treatment).

Gene_ID	Description	FPKM Leu	FPKM Rhi + Leu
NH16_RS04570	translation initiation factor IF-1	2937.34	7921.85
NH16_RS04590	DNA-directed RNA polymerase subunit alpha	2233.28	5548.51
NH16_RS02995	amino acid ABC transporter permease	1474.35	2727.61
NH16_RS02110	DUF1440 domain-containing protein	889.55	1634.84
NH16_RS08760	chorismate synthase	270.71	738.72
NH16_RS05800	UDP-N-acetylmuramoyl-tripeptide--D-alanyl-D- alanine ligase	240.80	527.74
NH16_RS07820	BglG family transcription antiterminator	174.48	20.57
NH16_RS00290	aldo/keto reductase	152.34	19.61
NH16_RS04090	FAD-dependent oxidoreductase	134.73	156.76
NH16_RS03055	23S rRNA (pseudouridine(1915)-N(3))-methyltransferase RlmH	29.97	26.73
NH16_RS07315	CCA tRNA nucleotidyltransferase	24.45	11.70
NH16_RS07865	hydroxyethylthiazole kinase	13.69	0.00
NH16_RS07350	YozE family protein	13.49	0.00
NH16_RS04800	D-alanine--D-alanine ligase	0.00	75.73

Table S6. Differentially expressed (mean normalized FPKM value) representative *Leuconostoc mesenteroides* (Leu) genes in the roots with high expression at 3-day post inoculation (dpi) of sugar beet alone or in combination with *Rhizoctonia solani* (Rhi). Data are Mean of 4 biological replicates ($p < 0.01$; mock vs. treatment).

Gene_ID	Description	FPKM Leu	FPKM Rhi + Leu
NH16_RS04635	50S ribosomal protein L13	3773.66	5770.22
NH16_RS04540	50S ribosomal protein L18	2458.58	2860.44
NH16_RS00880	50S ribosomal protein L20	2000.43	2528.66
NH16_RS02055	response regulator transcription factor	1611.50	1400.55
NH16_RS05935	ABC transporter substrate-binding protein	1206.72	581.07
NH16_RS02980	ABC transporter permease	1046.15	940.56
NH16_RS08775	shikimate dehydrogenase	502.90	978.61
NH16_RS03920	ATP-binding cassette domain-containing protein	439.07	413.49
NH16_RS01450	histidinol-phosphate transaminase	424.75	795.25
NH16_RS06140	GTPase ObgE	359.68	418.49
NH16_RS00635	mevalonate kinase	355.41	620.14
NH16_RS04910	ferrous iron transport protein B	318.99	223.13
NH16_RS05505	excinuclease ABC subunit UvrA	291.94	244.21
NH16_RS01265	primosomal protein N	224.63	188.66
NH16_RS03610	FAD-dependent oxidoreductase	138.08	105.74
NH16_RS02260	aromatic acid exporter family protein	108.81	100.65
NH16_RS02915	lactoylglutathione lyase	105.73	102.54
NH16_RS02510	Mini-ribonuclease 3	76.02	122.39
NH16_RS04190	amino acid ABC transporter ATP-binding protein	70.31	33.88
NH16_RS07305	o-succinylbenzoate synthase	57.41	87.07
NH16_RS01270	GNAT family N-acetyltransferase	48.57	33.64
NH16_RS07160	amidophosphoribosyltransferase	44.74	78.87
NH16_RS07145	phosphoribosylformylglycinamidine synthase subunit PurS	41.82	82.36
NH16_RS04400	serine--tRNA ligase	34.23	22.15
NH16_RS06635	ECF transporter S component	33.62	24.01
NH16_RS00970	phosphoketolase	29.12	66.56
NH16_RS01340	GNAT family N-acetyltransferase	28.65	17.06
NH16_RS07715	hypothetical protein	27.50	33.87
NH16_RS05700	DUF1275 domain-containing protein	23.89	21.39
NH16_RS01120	hypothetical protein	20.66	18.16
NH16_RS06285	acetolactate decarboxylase	13.48	9.82
NH16_RS02500	sigma-70 family RNA polymerase sigma factor	8.10	240.92
NH16_RS08260	citrate lyase acyl carrier protein	5.71	33.46
NH16_RS07280	hypothetical protein	1.38	349.45

Table S7. Sugar beet RNAseq read details.

Sample	Raw Data		Valid Data		Valid Ratio(reads)	Q20%	Q30%	GC content%
	Read	Base	Read	Base				
d1_Leu1	65487672	9.82G	60064640	9.01G	91.72	99.99	98.79	48
d1_Leu2	62645882	9.40G	60058724	9.01G	95.87	99.99	98.65	47
d1_Leu3	67739008	10.16G	62695848	9.40G	92.56	99.99	98.77	47.50
d1_Leu4	69125774	10.37G	63245072	9.49G	91.49	99.99	98.82	48
d1_M1	63901668	9.59G	60226154	9.03G	94.25	99.99	98.82	48
d1_M2	65165976	9.77G	61274562	9.19G	94.03	99.99	98.79	48
d1_M3	69461552	10.42G	65411518	9.81G	94.17	99.99	98.82	48.50
d1_M4	64456940	9.67G	60311376	9.05G	93.57	99.99	98.86	48.50
d1_Rhi1	68448048	10.27G	63212404	9.48G	92.35	99.99	98.81	48
d1_Rhi2	68536942	10.28G	65361098	9.80G	95.37	99.99	98.64	47
d1_Rhi3	66430266	9.96G	63280294	9.49G	95.26	99.99	98.72	47.50
d1_Rhi4	64294640	9.64G	60046890	9.01G	93.39	99.99	98.87	48
d1_Rhi_Leu1	67359690	10.10G	63339428	9.50G	94.03	99.99	98.70	47.50
d1_Rhi_Leu2	64813718	9.72G	61368888	9.21G	94.69	99.99	98.51	47
d1_Rhi_Leu3	68536094	10.28G	65091852	9.76G	94.97	99.99	98.68	47.50
d1_Rhi_Leu4	54656370	8.20G	52127104	7.82G	95.37	99.99	98.89	47
d2_Leu1	53075054	7.96G	48376236	7.26G	91.15	99.99	98.87	48.50
d2_Leu2	44465704	6.67G	37941492	5.69G	85.33	99.99	98.88	47
d2_Leu3	60519132	9.08G	55459260	8.32G	91.64	99.99	98.88	48.50
d2_Leu4	61772506	9.27G	55131028	8.27G	89.25	99.99	98.85	47
d2_M1	44341304	6.65G	41875280	6.28G	94.44	99.99	98.84	48
d2_M2	48503412	7.28G	45188244	6.78G	93.17	99.99	98.85	48.50
d2_M3	63920158	9.59G	60085890	9.01G	94.00	99.99	98.87	49
d2_M4	59143198	8.87G	55370898	8.31G	93.62	99.99	98.92	49.50
d2_Rhi1	55408084	8.31G	52154134	7.82G	94.13	99.99	98.78	48
d2_Rhi2	49712480	7.46G	47376110	7.11G	95.30	99.99	98.64	48
d2_Rhi3	51773938	7.77G	49823316	7.47G	96.23	99.99	98.71	47
d2_Rhi4	66055244	9.91G	59612038	8.94G	90.25	99.99	98.90	47
d2_Rhi_Leu1	61695938	9.25G	56658942	8.50G	91.84	99.99	98.72	48
d2_Rhi_Leu2	57304326	8.60G	51561444	7.73G	89.98	99.99	98.67	48
d2_Rhi_Leu3	59011124	8.85G	49066802	7.36G	83.15	99.99	98.82	48
d2_Rhi_Leu4	61023008	9.15G	58262790	8.74G	95.48	99.99	98.74	48
d3_Leu1	49083418	7.36G	47192444	7.08G	96.15	99.99	98.68	47
d3_Leu2	46993408	7.05G	45413038	6.81G	96.64	99.99	98.66	47
d3_Leu3	53948730	8.09G	51981732	7.80G	96.35	99.99	98.66	47
d3_Leu4	68769002	10.32G	66169798	9.93G	96.22	99.99	98.75	47
d3_M1	45436988	6.82G	43011480	6.45G	94.66	99.99	98.72	47.50
d3_M2	53431094	8.01G	52010420	7.80G	97.34	99.99	98.52	47
d3_M3	51522922	7.73G	49713912	7.46G	96.49	99.99	98.57	46
d3_M4	57084918	8.56G	55049850	8.26G	96.44	99.99	98.75	47
d3_Rhi1	57132750	8.57G	55350464	8.30G	96.88	99.99	98.53	47
d3_Rhi2	57301378	8.60G	55627196	8.34G	97.08	99.99	98.42	46
d3_Rhi3	46337168	6.95G	45154378	6.77G	97.45	99.99	98.52	46
d3_Rhi4	50122596	7.52G	48955106	7.34G	97.67	99.99	98.70	46
d3_Rhi_Leu1	55783156	8.37G	53886714	8.08G	96.60	99.99	98.63	48
d3_Rhi_Leu2	57412382	8.61G	55667746	8.35G	96.96	99.99	98.47	47
d3_Rhi_Leu3	53120722	7.97G	49507284	7.43G	93.20	99.99	98.69	47
d3_Rhi_Leu4	54374988	8.16G	52805640	7.92G	97.11	99.99	98.89	46

Table S8. *Rhizoctonia solani* RNAseq read details.

Sample	Raw Data		Valid Data		Valid Ratio(reads)	Q20%	Q30%	GC content%
	Read	Base	Read	Base				
d1_Leu1	64271026	9.64G	58948738	8.84G	91.72	99.99	98.79	48
d1_Leu2	62645882	9.40G	60058724	9.01G	95.87	99.99	98.65	47
d1_Leu3	69569854	10.44G	64385608	9.66G	92.55	99.99	98.77	47.50
d1_Leu4	66796924	10.02G	61118316	9.17G	91.50	99.99	98.82	48
d1_M1	64605246	9.69G	60887334	9.13G	94.25	99.99	98.82	48
d1_M2	66425612	9.96G	62456846	9.37G	94.03	99.99	98.79	48
d1_M3	67018674	10.05G	63111876	9.47G	94.17	99.99	98.82	48.50
d1_M4	69603366	10.44G	65118684	9.77G	93.56	99.99	98.86	48.50
d1_Rhi1	64571866	9.69G	59645378	8.95G	92.37	99.99	98.81	48
d1_Rhi2	69975860	10.50G	66730696	10.01G	95.36	99.99	98.64	47
d1_Rhi3	63610672	9.54G	60599716	9.09G	95.27	99.99	98.73	47.50
d1_Rhi4	69064436	10.36G	64497360	9.67G	93.39	99.99	98.87	48
d1_Rhi_Leu1	65978810	9.90G	62043514	9.31G	94.04	99.99	98.70	47.50
d1_Rhi_Leu2	68541860	10.28G	64890528	9.73G	94.67	99.99	98.51	47
d1_Rhi_Leu3	65055476	9.76G	61793902	9.27G	94.99	99.99	98.68	47.50
d1_Rhi_Leu4	54656370	8.20G	52127104	7.82G	95.37	99.99	98.89	47
d2_Leu1	53075054	7.96G	48376236	7.26G	91.15	99.99	98.87	48.50
d2_Leu2	44465704	6.67G	37941492	5.69G	85.33	99.99	98.88	47
d2_Leu3	60519132	9.08G	55459260	8.32G	91.64	99.99	98.88	48.50
d2_Leu4	61772506	9.27G	55131028	8.27G	89.25	99.99	98.85	47
d2_M1	44341304	6.65G	41875280	6.28G	94.44	99.99	98.84	48
d2_M2	48503412	7.28G	45188244	6.78G	93.17	99.99	98.85	48.50
d2_M3	69525488	10.43G	65344604	9.80G	93.99	99.99	98.87	49
d2_M4	59143198	8.87G	55370898	8.31G	93.62	99.99	98.92	49.50
d2_Rhi1	55408084	8.31G	52154134	7.82G	94.13	99.99	98.78	48
d2_Rhi2	49712480	7.46G	47376110	7.11G	95.30	99.99	98.64	48
d2_Rhi3	51773938	7.77G	49823316	7.47G	96.23	99.99	98.71	47
d2_Rhi4	63638294	9.55G	57437044	8.62G	90.26	99.99	98.90	47
d2_Rhi_Leu1	61695938	9.25G	56658942	8.50G	91.84	99.99	98.72	48
d2_Rhi_Leu2	57304326	8.60G	51561444	7.73G	89.98	99.99	98.67	48
d2_Rhi_Leu3	59011124	8.85G	49066802	7.36G	83.15	99.99	98.82	48
d2_Rhi_Leu4	61023008	9.15G	58262790	8.74G	95.48	99.99	98.74	48
d3_Leu1	49083418	7.36G	47192444	7.08G	96.15	99.99	98.68	47
d3_Leu2	46993408	7.05G	45413038	6.81G	96.64	99.99	98.66	47
d3_Leu3	53948730	8.09G	51981732	7.80G	96.35	99.99	98.66	47
d3_Leu4	66782970	10.02G	64261478	9.64G	96.22	99.99	98.75	47
d3_M1	45436988	6.82G	43011480	6.45G	94.66	99.99	98.72	47.50
d3_M2	53431094	8.01G	52010420	7.80G	97.34	99.99	98.52	47
d3_M3	51522922	7.73G	49713912	7.46G	96.49	99.99	98.57	46
d3_M4	57084918	8.56G	55049850	8.26G	96.44	99.99	98.75	47
d3_Rhi1	57132750	8.57G	55350464	8.30G	96.88	99.99	98.53	47
d3_Rhi2	57301378	8.60G	55627196	8.34G	97.08	99.99	98.42	46
d3_Rhi3	46337168	6.95G	45154378	6.77G	97.45	99.99	98.52	46
d3_Rhi4	50122596	7.52G	48955106	7.34G	97.67	99.99	98.70	46
d3_Rhi_Leu1	55783156	8.37G	53886714	8.08G	96.60	99.99	98.63	48
d3_Rhi_Leu2	57412382	8.61G	55667746	8.35G	96.96	99.99	98.47	47
d3_Rhi_Leu3	53120722	7.97G	49507284	7.43G	93.20	99.99	98.69	47
d3_Rhi_Leu4	54374988	8.16G	52805640	7.92G	97.11	99.99	98.89	46

Table S9. *Leuconostoc mesenteroides* RNAseq read details.

Sample	Raw Data		Valid Data		Valid Ratio(reads)	Q20%	Q30%	GC content%
	Read	Base	Read	Base				
d1_Leu1	67942720	10.19G	62309402	9.35G	91.71	99.99	98.79	48
d1_Leu2	62645882	9.40G	60058724	9.01G	95.87	99.99	98.65	47
d1_Leu3	66746314	10.01G	61778412	9.27G	92.56	99.99	98.77	47.50
d1_Leu4	68447286	10.27G	62626084	9.39G	91.50	99.99	98.82	48
d1_M1	64801402	9.72G	61071356	9.16G	94.24	99.99	98.82	48
d1_M2	66372462	9.96G	62406930	9.36G	94.03	99.99	98.79	48
d1_M3	65046252	9.76G	61258980	9.19G	94.18	99.99	98.82	48.50
d1_M4	63496436	9.52G	59413694	8.91G	93.57	99.99	98.86	48.50
d1_Rhi1	67333198	10.10G	62185678	9.33G	92.36	99.99	98.81	48
d1_Rhi2	67503644	10.13G	64377268	9.66G	95.37	99.99	98.64	47
d1_Rhi3	66430266	9.96G	63280294	9.49G	95.26	99.99	98.72	47.50
d1_Rhi4	66257120	9.94G	61877254	9.28G	93.39	99.99	98.87	48
d1_Rhi_Leu1	65227716	9.78G	61337848	9.20G	94.04	99.99	98.70	47.50
d1_Rhi_Leu2	68389360	10.26G	64746438	9.71G	94.67	99.99	98.51	47
d1_Rhi_Leu3	66198624	9.93G	62877744	9.43G	94.98	99.99	98.68	47.50
d1_Rhi_Leu4	54656370	8.20G	52127104	7.82G	95.37	99.99	98.89	47
d2_Leu1	53075054	7.96G	48376236	7.26G	91.15	99.99	98.87	48.50
d2_Leu2	44465704	6.67G	37941492	5.69G	85.33	99.99	98.88	47
d2_Leu3	60519132	9.08G	55459260	8.32G	91.64	99.99	98.88	48.50
d2_Leu4	61772506	9.27G	55131028	8.27G	89.25	99.99	98.85	47
d2_M1	44341304	6.65G	41875280	6.28G	94.44	99.99	98.84	48
d2_M2	48503412	7.28G	45188244	6.78G	93.17	99.99	98.85	48.50
d2_M3	63664040	9.55G	59844910	8.98G	94.00	99.99	98.87	49
d2_M4	59143198	8.87G	55370898	8.31G	93.62	99.99	98.92	49.50
d2_Rhi1	55408084	8.31G	52154134	7.82G	94.13	99.99	98.78	48
d2_Rhi2	49712480	7.46G	47376110	7.11G	95.30	99.99	98.64	48
d2_Rhi3	51773938	7.77G	49823316	7.47G	96.23	99.99	98.71	47
d2_Rhi4	66517092	9.98G	60026678	9.00G	90.24	99.99	98.90	47
d2_Rhi_Leu1	61695938	9.25G	56658942	8.50G	91.84	99.99	98.72	48
d2_Rhi_Leu2	57304326	8.60G	51561444	7.73G	89.98	99.99	98.67	48
d2_Rhi_Leu3	59011124	8.85G	49066802	7.36G	83.15	99.99	98.82	48
d2_Rhi_Leu4	61023008	9.15G	58262790	8.74G	95.48	99.99	98.74	48
d3_Leu1	49083418	7.36G	47192444	7.08G	96.15	99.99	98.68	47
d3_Leu2	46993408	7.05G	45413038	6.81G	96.64	99.99	98.66	47
d3_Leu3	53948730	8.09G	51981732	7.80G	96.35	99.99	98.66	47
d3_Leu4	67088486	10.06G	64554962	9.68G	96.22	99.99	98.75	47
d3_M1	45436988	6.82G	43011480	6.45G	94.66	99.99	98.72	47.50
d3_M2	53431094	8.01G	52010420	7.80G	97.34	99.99	98.52	47
d3_M3	51522922	7.73G	49713912	7.46G	96.49	99.99	98.57	46
d3_M4	57084918	8.56G	55049850	8.26G	96.44	99.99	98.75	47
d3_Rhi1	57132750	8.57G	55350464	8.30G	96.88	99.99	98.53	47
d3_Rhi2	57301378	8.60G	55627196	8.34G	97.08	99.99	98.42	46
d3_Rhi3	46337168	6.95G	45154378	6.77G	97.45	99.99	98.52	46
d3_Rhi4	50122596	7.52G	48955106	7.34G	97.67	99.99	98.70	46
d3_Rhi_Leu1	55783156	8.37G	53886714	8.08G	96.60	99.99	98.63	48
d3_Rhi_Leu2	57412382	8.61G	55667746	8.35G	96.96	99.99	98.47	47
d3_Rhi_Leu3	53120722	7.97G	49507284	7.43G	93.20	99.99	98.69	47
d3_Rhi_Leu4	54374988	8.16G	52805640	7.92G	97.11	99.99	98.89	46
